# Liver Transcriptome Responses to Heat Stress and Newcastle Disease Virus Infection in Genetically Distinct Chicken Inbred Lines

**DOI:** 10.3390/genes11091067

**Published:** 2020-09-11

**Authors:** Ying Wang, Perot Saelao, Colin Kern, Sihua Jin, Rodrigo A. Gallardo, Terra Kelly, Jack M. Dekkers, Susan J. Lamont, Huaijun Zhou

**Affiliations:** 1 Department of Animal Science, University of California, Davis, CA 95616, USA; ucywang@ucdavis.edu (Y.W.); psaelao@ucdavis.edu (P.S.); ckern@ucdavis.edu (C.K.); jsh3235@126.com (S.J.); 2 Feed the Future Innovation Lab for Genomics to Improve Poultry, University of California, Davis, CA 95616, USA; ragallardo@ucdavis.edu (R.A.G.); trkelly@ucdavis.edu (T.K.); jdekkers@iastate.edu (J.M.D.); sjlamont@iastate.edu (S.J.L.); 3 Department of Population Health and Reproduction, School of Veterinary Medicine, University of California, Davis, CA 95616, USA; 4 Department of Animal Science, Iowa State University, Ames, IA 50011, USA

**Keywords:** chicken, heat stress, Newcastle disease virus, RNA-seq

## Abstract

Heat stress results in reduced productivity, anorexia, and mortality in chickens. The objective of the study was to identify genes and signal pathways associated with heat stress and Newcastle disease virus (NDV) infection in the liver of chickens through RNA-seq analysis, using two highly inbred chicken lines (Leghorn and Fayoumi). All birds were held in the same environment until 14 days of age. On day 14, half the birds were exposed to 38 °C with 50% relative humidity for 4 h, then 35 °C until the end of the experiment. The remaining birds were kept at 25 °C throughout the experiment. The heat-treated birds were inoculated at 21 days of age with 10^7^ EID_50_ (One EID_50_ unit is the amount of virus that will infect 50 percent of inoculated embryos) NDV La Sota strain to investigate the effects of both heat stress and NDV infection. Physiological parameters were recorded as blood phenotypes at three stages: acute heat (AH), chronic heat (CH1), and chronic heat combined with NDV infection (CH&NDV), at 4 h, 7 days, and 10 days post-initiation of heat treatment, respectively. Our previous work revealed that the heat-resilient Fayoumi line maintained a more stable acid-base balance in their blood compared to the Leghorn line. Liver samples were harvested on both AH and CH&NDV to characterize the transcriptome profiles of these two inbred lines. Both genetic lines and treatments had large impact on the liver transcriptome. Fayoumi birds had more differentially expressed genes (DEGs) than Leghorn birds for both treatments. Metabolic and immune-related genes were on the DEG list, with Fayoumi having more immune-related DEGs than Leghorns, which was confirmed by gene functional enrichment analysis. Weighted correlation network analysis (WGCNA) indicated that the driver genes such as Solute Carrier Family genes could be very important for stabilizing the acid-base balance in Fayoumi birds during heat stress. Therefore, candidate genes such solute carrier family genes could be potential genetic targets that are regulated by Fayoumis to maintain physical hemostasis under heat stress. Differential gene expression showed that Leghorns mainly performed metabolic regulation in response to heat stress and NDV infection, while Fayoumis regulated both immune and metabolic functions. This study provides novel insights and enhances our understandings of liver response to heat stress of heat resilient and susceptible inbred chicken lines.

## 1. Introduction

All animals have a range of ambient temperatures that are appropriate to their physiological functions. When the ambient temperature increases above the upper critical range, animals start to suffer heat stress [1]. With global climate change, chickens may experience more heat stress during prolonged hot weather. Heat stress negatively impacts health and performance of poultry, from reduced growth and egg production to decreased meat and egg quality and safety [2,3], which results in significant economic loss for the US poultry industry and in developing countries such as those in Africa [4]. However, the cause for these deleterious effects on immunity and growth performance under heat stress is still poorly understood.

In response to heat stress, visible signs such as panting respiration, spreading wings, decreasing feed intake, drinking more, and shunting blood has been observed in chickens [2]. Panting increases respiratory rate, which causes respiratory alkalosis due to loss of carbon dioxide (CO_2_) and bicarbonate (HCO_3_) and results in an elevation of plasma pH [5]. Spreading wings helps to radiate heat from the body. Decreased food intake is one way to lower production of metabolic heat and to cool body temperature via the gut and intestinal tract [6]. Blood shunting away from gut is another way to reduce production of metabolic heat and increase heat loss [7]. The increase in water intake increases urine excretion and results in the loss of electrolyte balance [8]. 

It has been demonstrated that heat stress has negative impacts on the mammalian immune system by affecting both innate and adaptive immune responses [9]. Poultry immune responses will also be suppressed when exposed to heat stress, which can increase susceptibility to infectious diseases including viral infections such as Newcastle disease (ND) [10] that is considered the top one disease in poultry industry in developing countries [11]. Therefore, heat stress will increase the potential risk of infectious disease outbreaks just like continuous NDV epizootic in Africa [12]. 

Genetic background plays an important role in response to heat stress. Identification of genetic markers associated with heat resistance makes it possible to perform selective breeding for heat resilience in chickens. Characterization of genetically controlled physiological parameters that vary in response to heat stress will lay the foundation for utilizing these parameters as potential markers to select for heat stress resilience [13]. In our recent study, physiological parameters from two genetically distinct, highly inbred lines (Leghorn and Fayoumi) were measured and analyzed [14]. These two genetic lines responded differently during heat stress, especially at the acute heat stage. Leghorn birds had a significant acute response to heat and had significant electrolyte disruption, causing metabolic alkalosis. In contrast, Fayoumi birds were able to maintain electrolyte balance with respiratory alkalosis. It suggested that the acid-base balance was one of the key factors accounting for the genetic differences between these two lines. After sensing heat stress by the hypothalamus, the liver serves as a central organ for maintaining homeostasis and the overall metabolism by regulating fat, glucose and protein production [15]. Therefore, further molecular and cellular mechanistic investigation by liver transcriptome profiling is valuable to characterize gene expression alterations and reveal the complex genomic response to heat stress, which can provide more insights into the genetic regulation of heat tolerance in chickens. 

The current study is a part of the United States Agency for International Development (USAID) sponsored program Feed the Future Innovation Lab for Genomics to Improve Poultry, which aims to improve food security in Africa by enhancing resistance to Newcastle disease (ND) and heat stress in chickens (http//:gip.ucdavis.edu). The specific objective of this study was to identify novel genes and signaling pathways based on RNA-seq in the liver tissue that are associated with resilience to heat stress, utilizing the unique genetic lines of Fayoumi and Leghorn. The experimental design focused on heat stress combined with Newcastle disease virus (NDV) infection, which mimics the environment of village poultry in Africa.

## 2. Materials and Methods 

### 2.1. Experimental Populations and Design

The experimental design of this study has been described previously [14] and animal experiments were performed according to the guidelines approved by the Institutional Animal Care and Use Committee, University of California, Davis (IACUC #17853). In brief, chickens from two genetically highly inbred lines, Leghorn (GHs 6) and Fayoumi (M 15.2) from the Department of Animal Science, Iowa State University Poultry Farm (Ames, IA, USA) were used. One hundred and eleven birds, 55 Leghorns and 56 Fayoumis, were housed in two temperature and humidity-controlled isolators. Birds were provided with *ad libitum* access to food and water. On day 1, 30 Leghorn and 31 Fayoumi birds were randomly chosen as the treatment groups and housed in one isolator, while the remaining birds were used as non-treated groups in another isolator. The two lines were mixed in each isolator. The temperature and humidity settings for the two isolators were same from day 1 until day 14. From 14 days of age to the end of the experiment, the heat-treated groups were exposed to continuous heat stress of 38 °C for the first 4 h, and then decreased to 35 °C, while the non-treated groups were maintained at 25 °C throughout the whole experiment. On day 21, birds in the heat-treated groups were inoculated with 10^7^ EID_50_ Newcastle Disease virus (NDV) La Sota strain via the oculo-nasal route (~50 µL per nostril and eye). The non-treated birds were given 200 ul of phosphate buffered saline (PBS) using the same inoculation route.

### 2.2. Sample Collection and RNA Isolation

Liver tissues from 32 chickens (four individuals per genetic line per treatment) were collected for RNA isolation at 14 days of age (4 h post-heat-stress treatment, acute phase (AH)) and at 23 days of age (9 days post-heat-treatment, chronic phase 2 and 2 days post NDV inoculation (CH&NDV)), snap frozen in liquid nitrogen, and then stored at −80 °C until further use. Total RNA was isolated using Trizol (Invitrogen, Carlsbad, CA, USA) according to the manufacturer’s protocol. DNase I (Ambion, Austin, TX, USA) digestion was carried out after RNA isolation, and the RNA concentration and purity were determined by measuring absorbance at 260 nm and A260/A280 ratio using a NanoDrop ND-1000 spectrophotometer (NanoDrop Technologies, Wilmington, DE, USA). RNA quality was checked by Agilent Bioanalyzer (Agilent, Santa Clara, CA, USA). Extracted RNA was stored at −80 °C until further use.

### 2.3. RNA-seq Library Preparation

For each sample, 500 ng of total RNA was used to construct a strand specific cDNA library using NEBNext®Ultra RNA library prep Kit for Illumina® (New England BioLabs, Ipswich, MA, USA). The cDNA libraries were quantified by Qubit (ThermoFisher Scientific, Waltham, MA, USA) and validated by Agilent Bioanalyzer High Sensitivity DNA Assay (Agilent) and then sequenced on the HiSeq4000 platform (Illumina, San Diego, CA, USA) for 100 bp paired end reads with a minimum sequencing depth of 30 million reads (DNA Core Facility, University of California, Davis, Davis, CA, USA). Sequence data have been submitted through the Sequence Read Archive (https://www.ncbi.nlm.nih.gov/sra/) under accession number: SUB7442000.

### 2.4. RNA-seq Analysis

**Figure 1:**
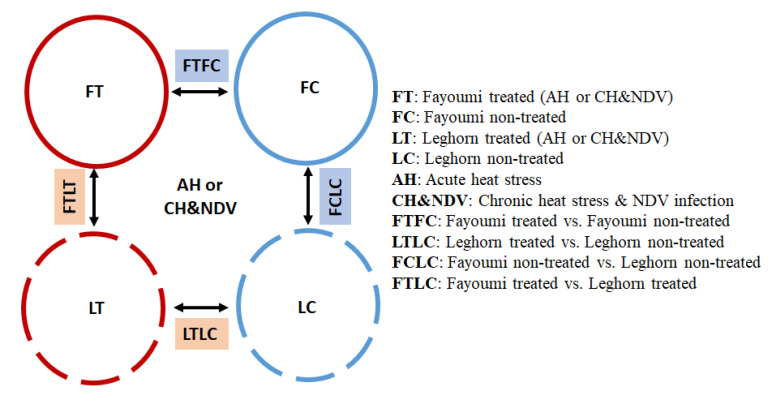
The comparison groups with acute heat stress and chronic heat stress & NDV infection for RNA-seq analysis.

Data from each treatment were analyzed separately. Two major factors were included for the RNA-seq analysis: condition (treated, non-treated) and line (Leghorn, Fayoumi), with four individuals for treatment by genetic line combination for each treatment. Raw reads were trimmed to remove adapter sequences and low-quality bases using the Trim Galore program (https://github.com/FelixKrueger/TrimGalore). TopHat [16] was used to align reads to the Gallus_gallus-6.0 reference genome downloaded from the Ensembl genome browser, and alignments were then filtered to remove those with a MAPQ alignment score of less than 15, which removes bad alignments and multi-mapped reads. Read counts for each transcript were generated from HTSeq. HTSeq counts were statistically analyzed using the R package DESeq2 to identify the differentially expressed genes (DEGs). The statistic model design included the effects of line and condition for each treatment, along with the interactions between condition and line. DEGs were determined if they had a false discovery rate (FDR) < 0.05. Gene expression profiles were compared between each pair of the 8 groups: Leghorn non-treated (LC) and Leghorn treated (LT) with AH and CH&NDV; Fayoumi non-treated (FC) and Fayoumi treated (FT) with AH and CH&NDV. The comparison groups are shown in Figure 1, in which FC vs. LC (FCLC) with AH or CH&NDV treatment and FT vs. LT (FTLT) with AH or CH&NDV treatment for between-line comparisons; LT vs. LC (LTLC) with AH or CH&NDV and FT vs. FC (FTFC) with AH or CH&NDV for within-line comparisons. For the between line comparisons, genes highly expressed in the Fayoumi line compared to the Leghorn line are Fayoumi-biased DEGs, while genes highly expressed in the Leghorn line compared to the Fayoumi line are Leghorn-biased DEGs.

### 2.5. Gene Ontology

Statistics related to overrepresentation of functional categories in the biological process of Gene Ontology and Kyoto Encyclopedia of Genes and Genomes (KEGG) pathways were performed using DAVID (The Database for Annotation, Visualization and Integrated Discovery) [17,18] by using DEG lists of each comparison. A *p*-value < 0.05, fold enrichment > 2 and FDR < 20% were considered as significant.

### 2.6. Pathway Analysis

Pathway analysis using the DEGs of within-line contrasts was performed by the Ingenuity Pathway Analysis software (IPA; Qiagen, Redwood City, CA, USA) [19]. Significant associations (*p*-value < 0.05) and a Z-score cutoff of |z| > 0 were used to identify significantly activated or inhibited pathways.

### 2.7. Gene Co-Expression Network Analysis

The Weighted Gene Co-expression Network Analysis (WGCNA) package in R was used for gene co-expression network analysis [20,21]. A soft threshold of 13 was set for generating an adjacency matrix based on co-expression and the minimum module size was 30. To evaluate associations of co-expressed gene clusters with line and treatment, the Leghorn and Fayoumi lines were given nominal values of 1 and 2 and non-treated and treated the nominal values of 0 and 1. For the continuous traits, association of co-expressed gene clusters with the continuous traits pH, carbon dioxide partial pressure (PCO_2_), bicarbonate (HCO_3_), base excess (BE), oxygen partial pressure (PO_2_), oxygen situation (sO_2_), glucose (Glu), sodium (Na^+^), potassium (K^+^) and ionized calcium (iCa^2+^) were also evaluated. The driver genes were identified by high absolute values of gene significance (GS > 0.5) and module membership (MM > 0.5) with a threshold of *p*-value < 0.05 [22]. 

## 3. Results

### 3.1. Effects of Heat Stress and NDV Infection on Gene Expression

In the current liver transcriptome profiles, of the 24,357 annotated chicken genes in the chicken Galgal 6.0 database, more than 77% of the annotated genes were identified in Leghorn, and 78% were identified in Fayoumi birds. The detailed mapping statistics are listed in Table 1. 

**Figure 2:**
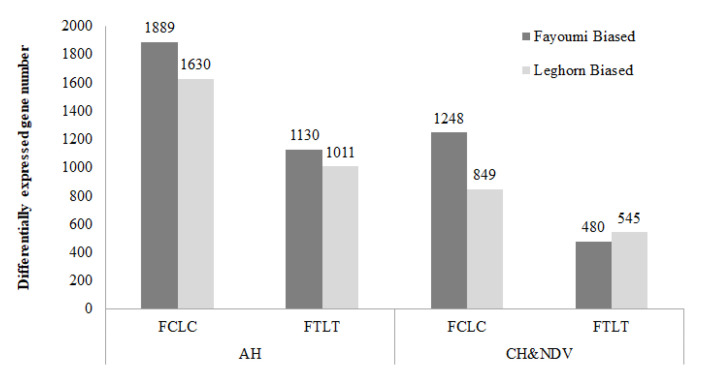
Numbers of differentially expressed gene identified between genetic lines. A false discovery rate (FDR) < 0.05 was used to classify genes as significantly differentially expressed. AH: Acute heat stress; CH&NDV: Chronic heat stress and NDV infection at 2 dpi; FCLC: Fayoumi non-treated vs. Leghorn non-treated; FTLT: Fayoumi treated vs. Leghorn treated.

**Figure 3:**
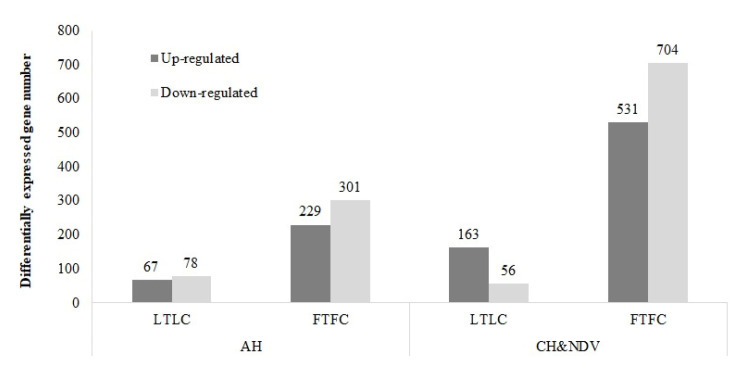
Numbers of differentially expressed gene identified within genetic lines. A false discovery rate < 0.05 was used to classify genes as significantly differentially expressed. AH: Acute heat stress; CH&NDV: Chronic heat stress and NDV infection at 2 dpi; LTLC: Leghorn treated vs. non-treated; FTFC: Fayoumi treated vs. non-treated.

Gene expression profiles were compared between each pair of the eight groups described in the method part as shown in Figure 1. There was no significant DEGs for the interaction effect. The number of DEGs for between and within-line comparisons are shown in Figure 2 and Figure 3.

#### 3.1.1. Between Genetic Lines

**Figure 4:**
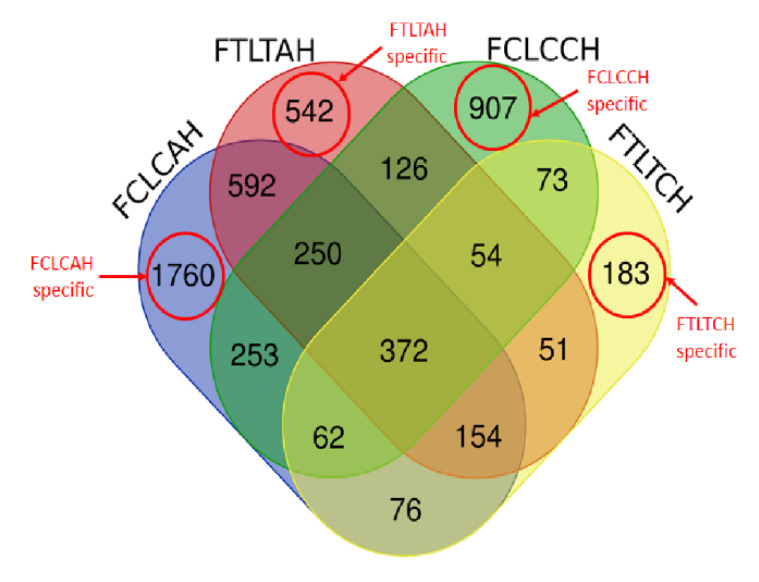
Venn diagram of differentially expressed genes between genetic lines. FCLCAH: Fayoumi non-treated vs. Leghorn non-treated with acute heat stress; FTLTAH: Fayoumi treated vs. Leghorn treated with acute heat stress; FCLCCH: Fayoumi non-treated vs. Leghorn non-treated with chronic heat stress and 2 dpi NDV infection; FTLTCH: Fayoumi treated vs. Leghorn treated with chronic heat stress and 2 dpi NDV infection. FCLCAH specific: specific DEGs in FCLCAH; FTLTAH specific: specific DEGs in FTLTAH; FCLCCH specific: specific DEGs in FCLCCH; FTLTCH specific: specific DEGs in FTLTCH.

Four between-line comparisons were analyzed (Figure 2). More DEGs of the between-line comparisons were Fayoumi-biased genes except the comparison of FTLT with CH&NDV having more Leghorn-biased genes. More DEGs had been identified at the early time point (day 14 or AH) than the later time point (day 23 or CH&NDV). There were 372 genes shared by all four comparisons shown in Figure 4, however, all these DEGs had same regulatory directions with treatments. There were 1760 DEGs specifically identified in the FCLC with AH, 542 DEGs in the FLTL with AH, 907 DEGs in the FCLC with CH&NDV, and 183 DEGs in the FTLT with CH&NDV (Figure 4). Detailed gene information and fold changes in each comparison are listed in Supplementary File 1.

#### 3.1.2. Within Genetic Lines

**Figure 5:**
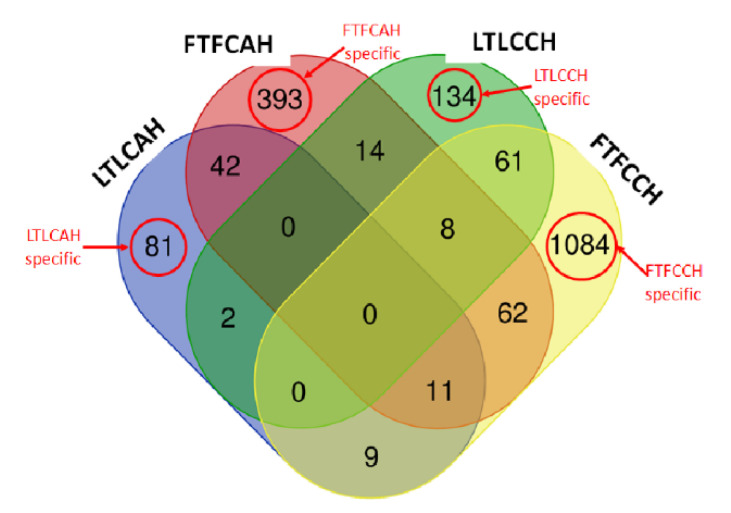
Venn diagram of differentially expressed genes within genetic lines. LTLCAH: Leghorn treated vs. non-treated with acute heat stress; FTFCAH: Fayoumi treated vs. non-treated with acute heat stress; LTLCCH: Leghorn treated vs. non-treated with chronic heat stress and 2 dpi NDV infection; FTFCCH: Fayoumi treated vs. non-treated with chronic heat stress and 2 dpi NDV infection. LTLCAH specific: specific DEGs in LTLCAH; FTFCAH specific: specific DEGs in LTLCAH; LTLCCH specific: specific DEGs in LTLCCH; FTFCCH specific: specific DEGs in FTFCCH.

When comparing the non-treated versus the treated birds within each genetic line, Leghorn birds had many fewer DEGs than Fayoumi birds at both AH and CH&NDV stages (Figure 3). More DEGs were down-regulated with the treatments in both genetic lines except Leghorn birds with CH&NDV in which more up-regulated DEGs were identified. There were no DEGs shared by four within-line comparisons (Figure 5). Leghorn and Fayoumi shared 53 DEGs with AH and 69 DEGs with CH&NDV. However, these DEGs had same regulation directions for both lines. Only two genes were consistently differentially expressed in Leghorn birds at AH and CH&NDV. One of them was a LncRNA gene and the other was DEAD-box helicase 55 (DDX55). DDX55 is a protein coding gene potentially involved in influenza A life cycle [23]. It was down-regulated at AH while up-regulated with CH&NDV treatment in Leghorns. Eighty-one DEGs were identified in Fayoumi birds and shared by both AH and CH&NDV treatment, in which eight DEGs, such as MX dynamin like GTPase 1 (MX1), T cell leukemia homebox 1 (TLX1) and heat shock protein family B (small) member 9 (HSPB9), had oppositely regulatory directions between Fayoumi AH and Fayoumi CH&NDV (Table 2). Detailed gene information and fold changes for these four comparisons are listed in Supplementary File 2.

### 3.2. Functional Categories of Differentially Expressed Genes

Gene ontology (GO) analysis was used to identify the function of these DEGs and the biological processes that they regulated. All DEGs in FCLC, FTLT, LTLC and FTFC at AH and CH&NDV were performed by functional enrichment analysis through the DAVID software (DAVID Bioinformatics Resources 6.8). The biological processes and KEGG pathways are presented as functional clusters.

#### 3.2.1. Between Genetic Lines

**Figure 6:**
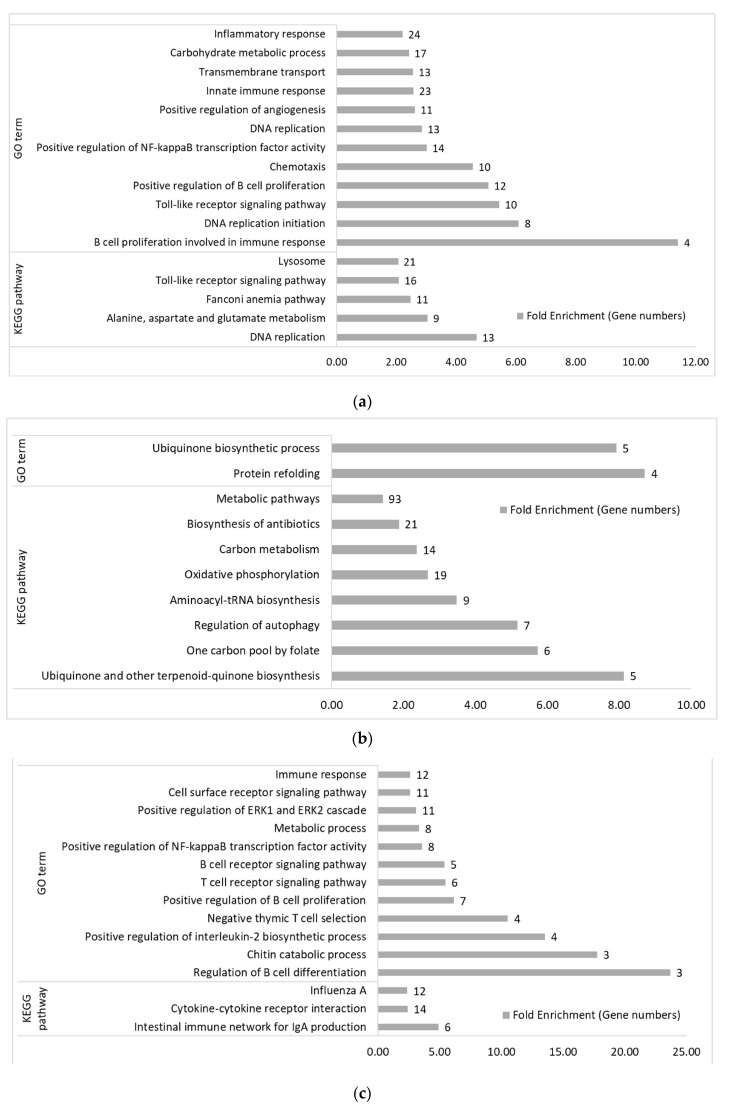

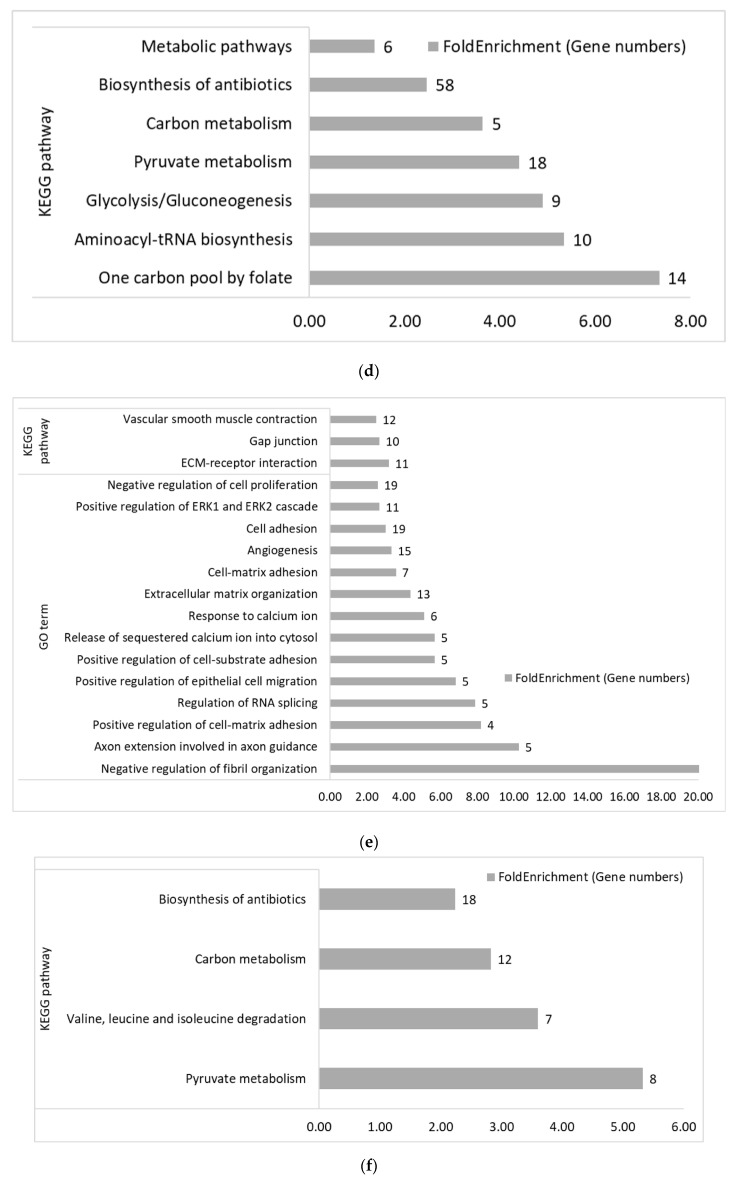

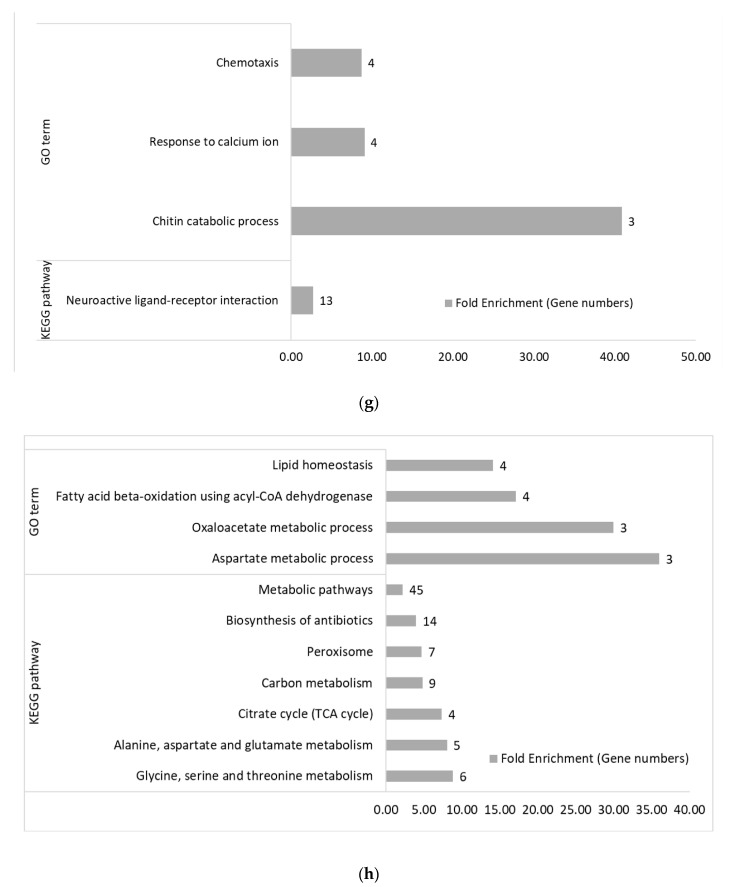
Gene ontology (GO) biological processes and KEGG pathway overrepresentation (*p* < 0.05, fold enrichment > 2 and FDR < 0.2) for between-line comparisons. (**a**) GO terms and KEGG pathways significantly enriched by Fayoumi-biased genes in the FCLC comparison at AH. (**b**) GO terms and KEGG pathways significantly enriched by Leghorn-biased genes in the FCLC comparison at AH. (**c**) GO terms and KEGG pathways significantly enriched by Fayoumi-biased genes in the FTLT comparison at AH. (**d**) KEGG pathways significantly enriched by Leghorn-biased genes in the FTLT comparison at AH. (**e**) GO terms and KEGG pathways significantly enriched by Fayoumi-biased genes in the FCLC comparison at CH&NDV. (**f**) GO terms and KEGG pathways significantly enriched by Leghorn-biased genes in the FCLC comparison at CH&NDV. (**g**) GO terms and KEGG pathways significantly enriched by Fayoumi-biased genes in the FTLT comparison at CH&NDV. (**h**) GO terms and KEGG pathways significantly enriched by Leghorn-biased genes in the FTLT comparison at CH&NDV.

On day 14, without treatment, Fayoumi-biased genes were significantly enriched in twelve GO terms and five KEGG pathways mainly involved in immune response and metabolic functions (Figure 6a), while Leghorn-biased genes had two GO terms and eight KEGG pathways which mostly involved in metabolic functions (Figure 6b). At the same time, with acute heat stress, Fayoumi-biased genes significantly enriched twelve GO terms and three KEGG pathways, which were mainly related with immune response (Figure 6c). No GO terms were identified but seven KEGG pathways were significantly enriched in the Leghorn-biased genes with AH treatment, in which, majority of them were metabolism-related (Figure 6d). Genes highly expressed in Fayoumi lines contributed more to immune functions, while genes highly expressed in Leghorn lines were mostly associated with metabolic functions at the acute heat stress stage. On day 21, when comparing the two genetic lines without treatment, twelve GO terms and three KEGG pathways were enriched by Fayoumi-biased genes (Figure 6e) and four KEGG pathways were enriched by Leghorn-biased genes (Figure 6f). Fayoumi-biased genes were more associated with cell regulation and ion transportation, while Leghorn-biased genes contributed more to Pyruvate and Carbon metabolism, and Biosynthesis of antibiotics. With the treatment of chronic heat stress and NDV infection, Fayoumi-biased genes were enriched in less GO terms (3) and KEGG pathways (1) (Figure 6g), however, Leghorn-biased genes were enriched in more GO terms (4) and KEGG pathways (7) (Figure 6h). The “Biosysnthesis of antibiotics” pathway was enriched by Leghorn-biased genes again with the combined treatment. The remaining GO terms and KEGG pathways enriched by between-line DEGs were metabolism-related.

Of the 382 overlapped genes across all 4 comparisons between genetic lines during AH and CH&NDV treatments (Figure 4), two KEGG pathways: Alanine, aspartate and glutamate metabolism and Ligand-receptor interaction were significantly enriched. For the 1760 FCLC specific genes at the AH stage (Figure 4), twelve GO terms and three KEGG pathways were significantly enriched by Fayoumi-biased genes, which were mostly involved in immune functions. One GO term (Ubiquinone biosynthetic process) and three KEGG pathways including Oxidative phosphorylation and Metabolic pathways were significantly enriched by Leghorn-biased genes. With AH treatment, the FTLT specific genes significantly enriched the T cell receptor signaling pathway and the Cytokine-cytokine receptor interaction by Fayoumi-biased genes. Four KEGG pathways were significantly enriched by the Leghorn-biased genes, which were all metabolism-related. At the later CH&NDV stage, without treatment, 907 FCLC specific genes (Figure 4) were significantly enriched in ten GO terms and one KEGG pathways by Fayoumi-biased genes and most of them were involved in immune functions. There was only one KEGG pathway “Drug metabolism” significantly enriched by Leghorn-biased genes. With the CH&NDV treatment, among the 183 FTLT specific genes (Figure 4), we only observed one GO term and five KEGG pathways significantly enriched by Leghorn-biased genes and these GO term and pathways were all metabolism associated. All the detailed GO terms and KEGG pathways are listed in Supplementary File 3.

#### 3.2.2. Within Genetic Lines

**Figure 7:**
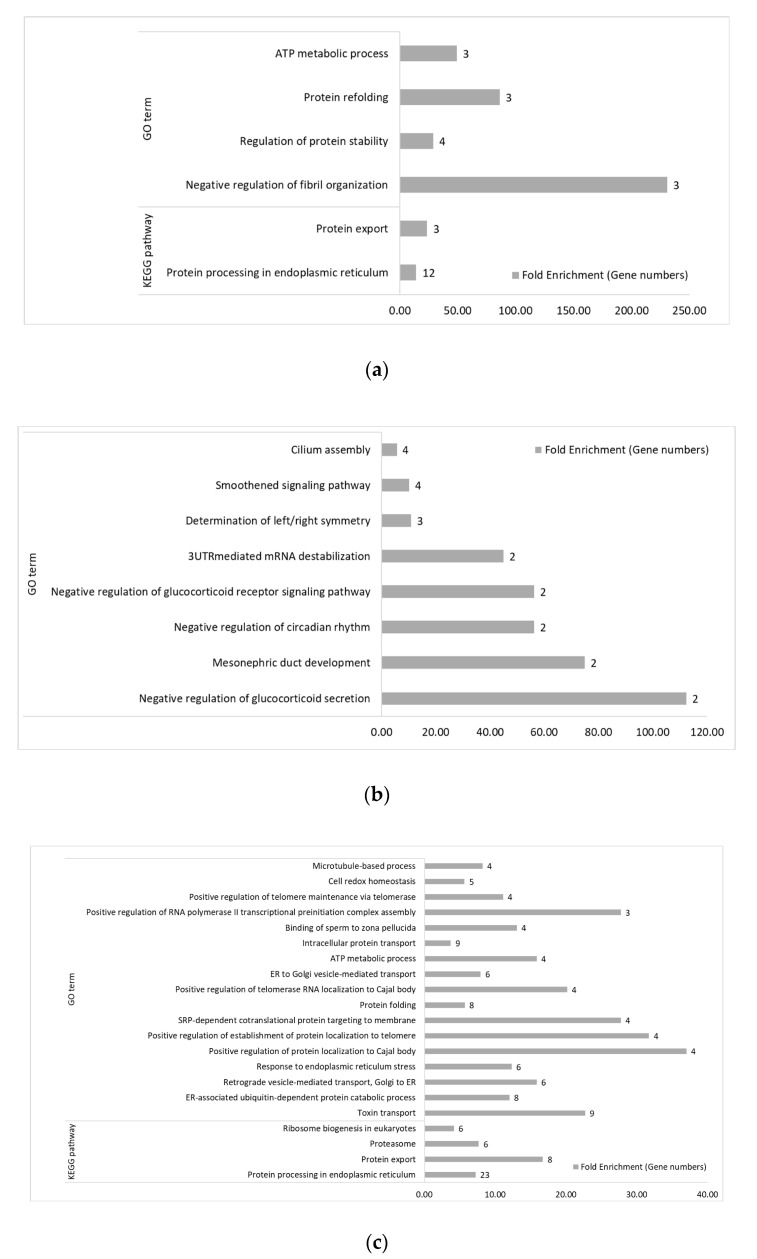

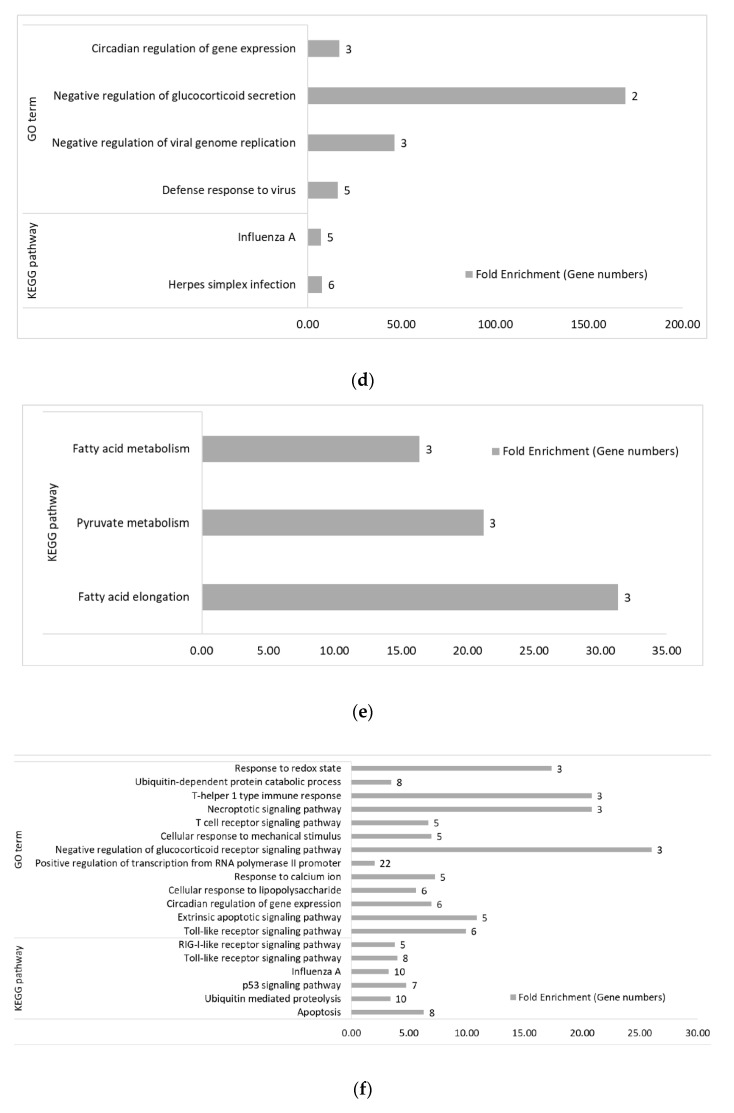

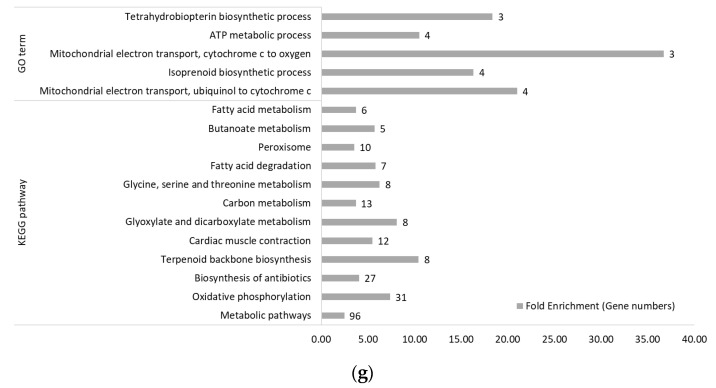
Gene ontology (GO) biological processes and KEGG pathway overrepresentation (*p* < 0.05, fold enrichment > 2 and FDR < 0.2) for within-line comparisons. (**a**) GO terms and KEGG pathways significantly enriched by down-regulated genes in the LTLC comparison at AH. (**b**) GO terms and KEGG pathways significantly enriched by up-regulated genes in the FTFC comparison at AH. (**c**) GO terms and KEGG pathways significantly enriched by down-regulated genes in the FTFC comparison at AH. (**d**) GO terms and KEGG pathways enriched by up-regulated genes in the LTLC comparison at CH&NDV. (**e**) GO terms and KEGG pathways significantly enriched by down-regulated genes in the LTLC comparison at CH&NDV. (**f**) GO terms and KEGG pathways significantly enriched by up-regulated genes in the FTFC comparison at CH&NDV. (**g**) GO terms and KEGG pathways significantly enriched by down-regulated genes in the FTFC comparison at CH&NDV.

Comparing the treated with the non-treated group within lines, the two genetic lines showed significantly distinct enriched biological function by GO terms and pathways. With AH treatment, no functional terms and pathways were significantly enriched in Leghorn up-regulated genes, which might be due to the fewer number of DEGs. Four GO terms and two KEGG pathways were significantly enriched by the down-regulated Leghorn genes, which were involved in protein folding, stability and export and ATP metabolic process (Figure 7a). In the Fayoumi birds, with AH treatment, eight GO terms were significantly enriched by the up-regulated genes including Negative regulation of glucocorticoid secretion and glucocorticoid receptor signaling pathway (Figure 7b). For the down-regulated genes with AH treatment, seventeen GO terms and four KEGG pathways were significantly enriched (Figure 7c). These terms and pathways were mostly related with cell localization, protein folding and transportation and ATP metabolic process.

With CH&NDV treatment, in Leghorns, viral infection associated GO terms and pathways, such as Defense response to virus, Negative regulation of viral genome replication and Influenza A pathway, were significantly enriched by the up-regulated genes (Figure 7d) and three metabolism-related KEGG pathways, fatty acid and pyruvate metabolism-related, were significantly enriched by the down-regulated genes (Figure 7e). In Fayoumi birds with CH&NDV, thirteen GO terms and six KEGG pathways were significantly enriched by the up-regulated genes (Figure 7f). Half of the GO terms were immune-related, and the other half were metabolism-related. For KEGG pathways, the majority was immune-related. There were five GO terms and twelve KEGG pathways significantly enriched by the down-regulated genes in Fayoumis (Figure 7g). All of these GO terms and pathways were metabolism-related. 

With AH treatment, one GO term “Response to stress” was significantly enriched in the Leghorn specific DEGs. Ten GO terms and four KEGG pathways were significantly enriched by the Fayoumi specific DEGs. With CH&NDV treatment, no GO terms or KEGG pathways were significantly enriched by Leghorn specific DEGs even with a higher DEG number (134 genes, Figure 5). In the Fayoumi line, eight GO terms and eleven KEGG pathways were significantly enriched by 1084 Fayoumi specific DEGs. These terms and pathways generally agreed with those ones significantly enriched by the down-regulated genes with CH&NDV treatment in Fayoumi birds. All GO terms and KEGG pathways are listed in Supplementary File 4. 

### 3.3. Significant Canonical Pathways Identified in Each Genetic Line with Treatments

**Figure 8:**
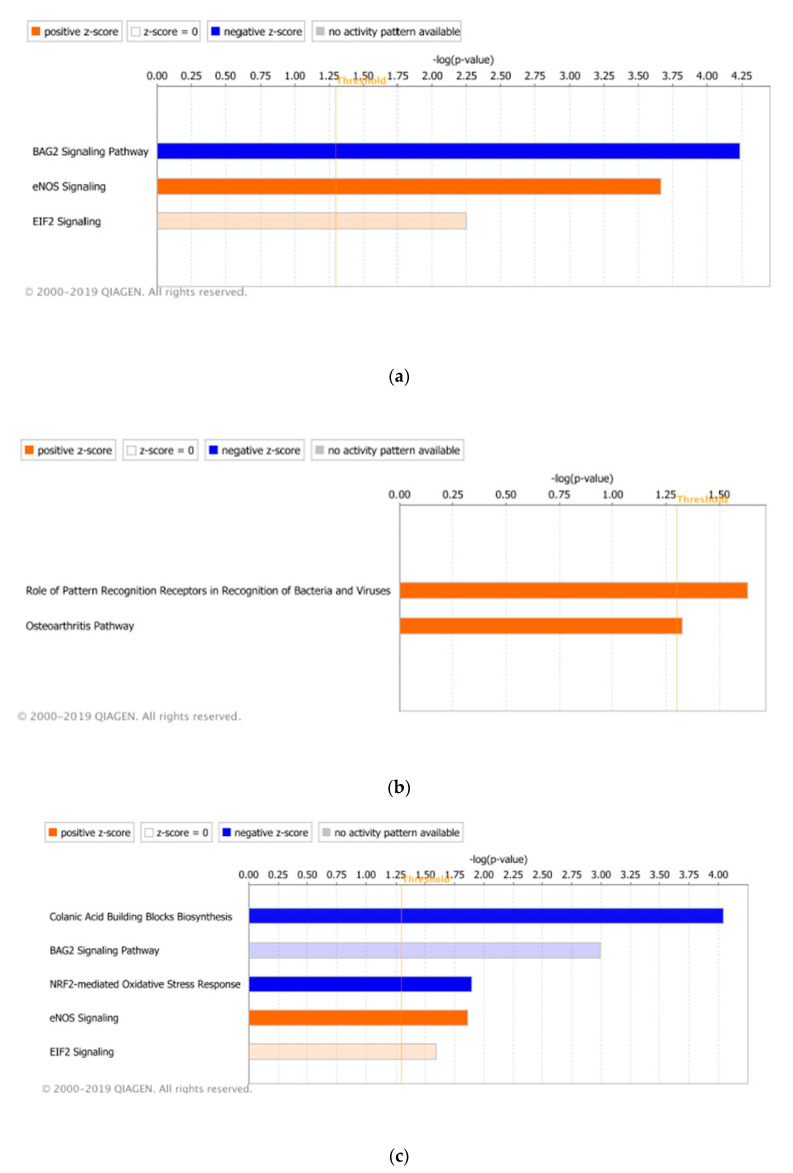

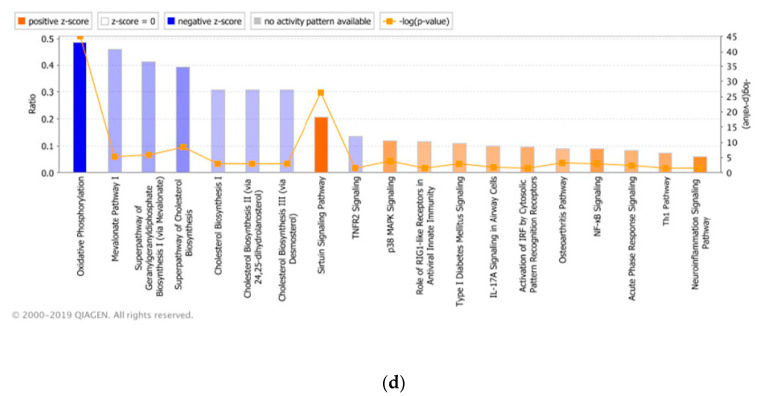
Significantly enriched canonical pathways through Ingenuity Pathway Analysis among differentially expressed genes by genetic line and treatment (*p* < 0.05 and z > 0), where orange (positive z-score) refers to predicted of activation and blue (negative z-score) predicted of inhibition. (**a**) Canonical pathways predicted in the Leghorn line with AH treatment. (**b**) Canonical pathways predicted in the Leghorn line with CH&NDV treatment. (**c**) Canonical pathways predicted in the Fayoumi line with AH treatment. (**d**) Top 10 Canonical pathways predicted in the Fayoumi line with CH&NDV treatment.

Pathway analysis of the DEGs identified between treated and non-treated birds in the two genetic lines was utilized to predict canonical pathways that were significantly enriched with the AH or CH&NDV treatment. For the AH treatment in the Leghorn line, three pathways: BAG2 Signaling Pathway (inhibited), eNOS Signaling (activated) and EIF2 Signaling (activated), were significantly enriched (Figure 8a). With CH&NDV treatment, two pathways (Role of Pattern Recognition Receptors in Recognition of Bacteria and Viruses and Osteoarthritis pathway) were significantly enriched in the Leghorn line (Figure 8b). For Fayoumi birds, five pathways were significantly enriched with the AH treatment (Figure 8c), in which three out of five were overlapped with Leghorn birds with the same stage of treatment. 

Colanic Acid Binding Blocks Biosynthesis (inhibited) and NRF2-mediated Oxidative Stress Response (inhibited) were the two specific pathways only identified in Fayoumi birds with AH treatment. Seventy-nine canonical pathways were significantly enriched by Fayoumi DEGs with CH&NDV treatment. The top significant pathways are shown in Figure 7d and the detailed pathway information is listed in Supplementary File 5. The most significantly activated pathway in Fayoumi birds at CH&NDV was Sirtuin Signaling Pathway and the most significantly inhibited pathway was Oxidative Phosphorylation. Other than metabolically related pathways such as Super pathway of Cholesterol Biosynthesis, Mevalonate Pathway I, Fatty Acid β-oxidation I and PPARα/RXRα Activation, many immune-related canonical pathways were significantly enriched in Fayoumi birds including Role of RIG-I like Receptors in Antiviral Innate Immunity, NF-ΚB Signaling, Toll-like Receptor Signaling and IL-6 Signaling.

### 3.4. Weighted Gene Co-Expression Network Analysis (WGCNA)

**Figure 9:**
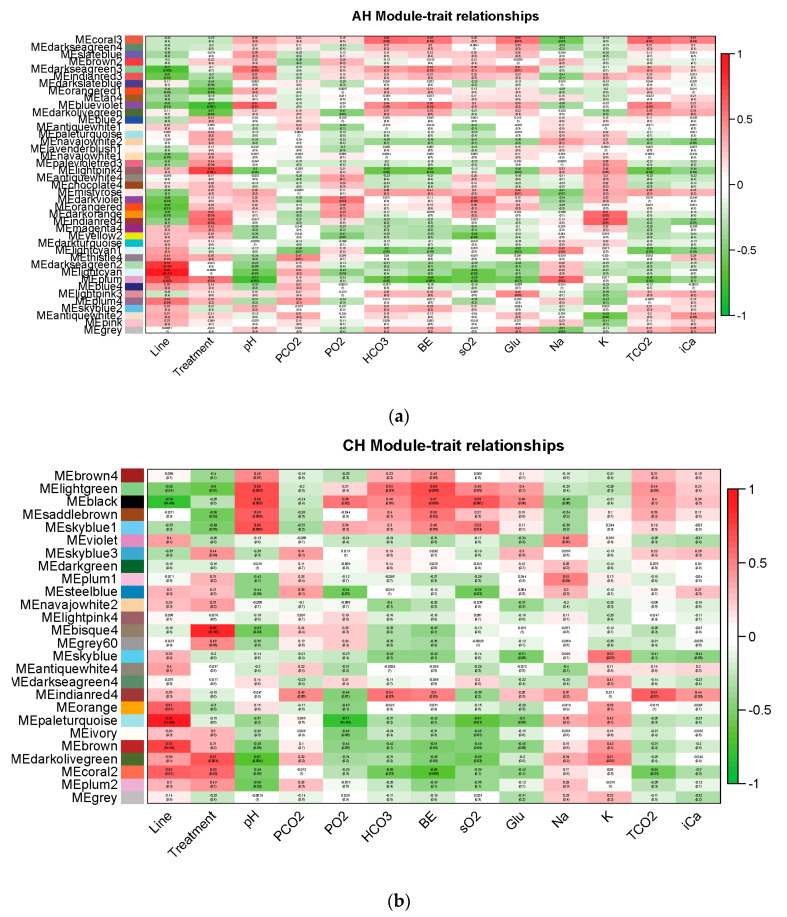
Module-trait relationships from WGNCA. Each module (*y*-axis) is correlated with each phenotype (*x*-axis) and the correlation and *p*-values were reported for each comparison. Strong positive correlations are colored in red, and strong negative correlations are colored in green. (**a**) Acute heat stress (**b**) Chronic heat stress and NDV infection.

To further explore potential underlying heat tolerance mechanisms, factors of interests such as line, treatment and physiological parameters related to heat stress measured in blood were used to identify potential important driver genes using the WGCNA method. Data were separately analyzed by treatment type: AH and CH&NDV. Normalized counts of all genes were clustered into modules based on similarities in gene expression with either AH or CH&NDV treatment (Figure 9a,b). Forty-one gene modules were identified with AH and twenty-six modules were identified with CH&NDV treatment. The correlations of these gene modules with line, treatment or physiological parameters associated with heat stress were further calculated. Significantly correlated gene modules with factors were summarized in Table 3. For the line effect, the positive correlation indicated higher gene expression in Fayoumi birds than Leghorns in the correlated gene modules. For the treatment effect, the positive correlation indicated higher gene expression in the treated group than the non-treated group. Some gene modules were correlated with more than one factor in the correlated gene modules. As expected for closely related traits such as blood gases (pH, PCO_2_, TCO_2_, HCO_3_, BE and PO_2_, sO_2_) and electrolytes (Na^+^, K^+^, iCa^2+^ and Glu), their correlated gene modules were relatively consistent (Table 4 and Table 5). Gene modules positively correlated with line (genes had higher expression levels in Fayoumis than Leghorns) were always negatively correlated with PO_2_ and sO_2_ with both AH and CH&NDV treatments. With AH, the Lightpink4 module, which was positively correlated with treatment (Genes had higher expression levels with the treatment), was negatively correlated with TCO_2_ and HCO_3_. The Blueviolet module, which was negatively correlated with the AH treatment (Genes had higher expression levels in the non-treated group), was positively correlated with pH and BE. With CH&NDV treatment, the Lightgreen module was negatively correlated with the treatment but positively correlated with pH, HCO_3_ and BE.

Furthermore, driver genes within the significantly correlated gene modules were identified. The top five annotated genes with the highest absolute gene significance (GS) for a factor and absolute value module membership (MM) are presented in Table 6 and Table 7. More detailed information is listed in Supplementary File 6. RAD52 motif containing 1 (RDM1) and Chitinase acidic (CHIA) were always the top 1 and 2 driver genes in the Lightcyan module at AH and in the Paleturquoise module at CH&NDV. Many metabolic and heat stress related genes were identified as driver genes at AH. Heat shock protein family A (Hsp70) member 5 (HSPA5) gene was identified in the Blueviolet module which was negatively correlated with the treatment and had a higher expression level in the non-treated group. Heat shock protein 90 α family class A member1 and class B member 1 (HSP90AA1 and HSP90AB1) were identified in the pH correlated gene modules (Blueviolet and Plum modules). Solute carrier family 24 member 1 (SLC24A1) was identified in the Coral3 module which were positively correlated with several blood gas phenotypes such as HCO3, TCO_2_, BE and negatively correlated with the Na+ level. Potassium calcium-activated channel subfamily M regulatory β subunit 4 (KCNMB4) was shown in the Antiquewhite2 module which was negatively correlated with the blood K^+^ level. With CH&NDV, besides the metabolism associated genes, immune-related genes were identified as driver genes as well. MX1 gene, previously reported having antiviral activities in many studies [19,24], was identified as one of the driver genes in the Bisque4 module which was positively correlated with CH&NDV treatment and had a higher expression level with the treatment. Interferon induced with helicase domain 1 (IFIH1) gene was identified in the Lightgreen module that negatively correlated with CH&NDV treatment and had a higher expression level in the non-treated group.

GO terms from the biological process and KEGG pathways were significantly enriched by using the entire gene group within significantly correlated gene modules with traits (Supplementary File 7). Genes in the significantly correlated gene modules at AH were more involved in proteasome, protein metabolism and cell localization. With CH&NDV treatment, genes in the significantly correlated gene modules were identified that not only contributed to metabolic functions such as fatty acid metabolism and metabolic pathways, but also played roles in immune functions, for example, the biosynthesis of antibiotics pathway and GO terms: negative regulation of apoptotic process and T-helper 1 type immune response were significantly enriched.

## 4. Discussion

Global transcriptome analysis of the host response to NDV infection under heat stress has been studied by our group in two major tissues where NDV primarily replicates i.e., the harderian gland and the lung by using the same individual birds from the same experiment of the current study [25,26]. From the results, a more activated immune response was observed in Fayoumis than Leghorns at two days post-infection with overall lower viral titer, indicating that Fayoumis were relatively more resistant to NDV infection compared to the Leghorns under heat stress [25,26]. As a part of the Feed the Future Innovation Lab Genomics to Improve Poultry (GIP) program, the presented study focuses on investigating the host response to the combination of heat stress and NDV infection that are commonly experienced by backyard poultry flocks in African countries. Previous findings on the physiological responses to heat stress in these two lines showed that Fayoumis were more heat resistant and Leghorns were more susceptible to heat stress based on measurements of blood parameters [14]. It is well known that liver plays important roles in maintaining homeostasis when facing heat stress by controlling blood metabolites levels such as sugars, lipids and amino acids [27]. Therefore, the phenotypical response due to heat stress we observed previously could be a consequence of the liver regulation. This comprehensive liver transcriptome analysis is able to provide additional insights into underlying molecular mechanisms of heat stress response, therefore contributing to knowledge needed to make genetic improvement of chickens by adapting to high ambient temperatures in Africa. The current study was the first to investigate host response to both acute heat stress and chronic heat stress combined with NDV infection in the two distinct highly inbred chicken lines. 

### 4.1. Host Response to Acute Heat Stress

#### 4.1.1. The Leghorn line

Down-regulated DEGs in Leghorns significantly enriched energy production regulation and protein processing and transportation functional terms. Down-regulation of four heat shock family genes (HSP99AA1, HSPA2, HSPA5 and HSPA8) with AH (Table 8), led the inhibition of the BAG2 signaling pathway. 

**Figure 10:**
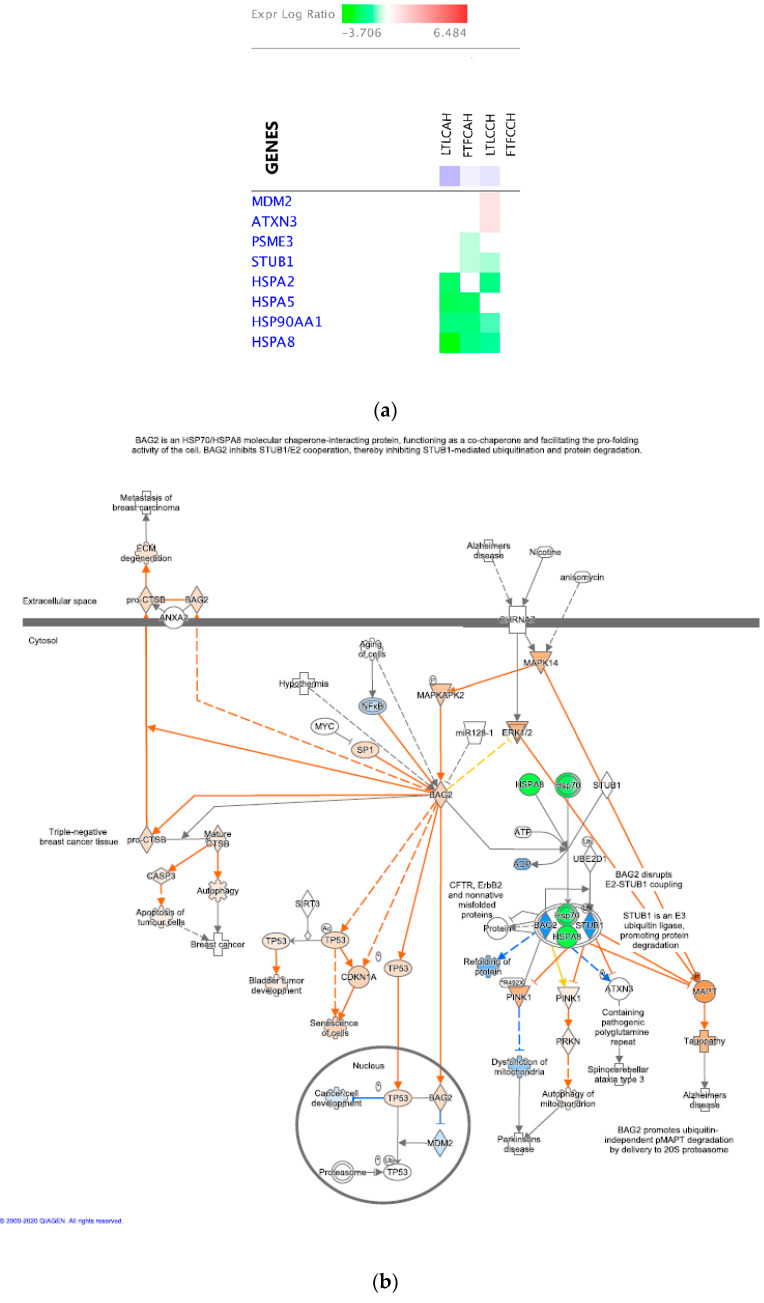

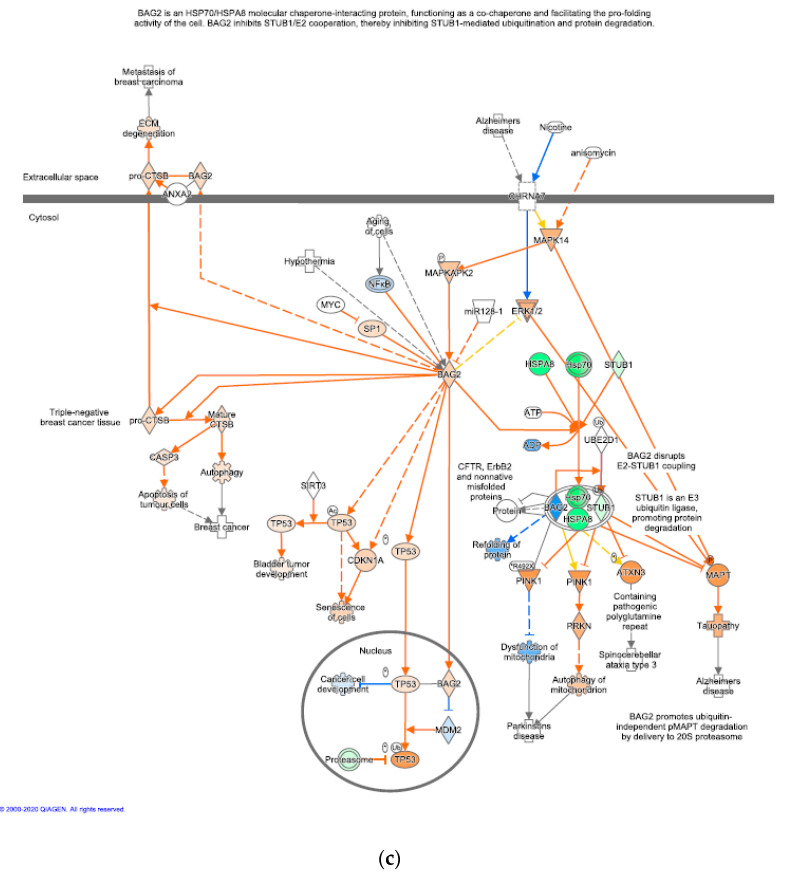
BAG2 signaling pathway and gene heat map in within-line comparisons with acute heat stress. (**a**) The DEG heatmap matching to the pathway; (**b**) The molecule activity prediction of the pathway in Leghorn birds with AH; (**c**) The molecule activity prediction of the pathway in Fayoumi birds with AH.

BAG2 is an important pro-folding regulator of protein triage by interacting with HSPA70/HSPA8 molecular chaperones [28]. Inhibiting this pathway and then inhibiting refolding of misfolded proteins (Figure 10b) might potentially induce more protein degradation, which may make Leghorn birds to be more susceptible to heat stress.

#### 4.1.2. The Fayoumi Line

There are many metabolic-related genes identified as DEGs with the AH treatment in Fayoumis (Table 9). The BAG2 signaling pathway was also predicted to be inhibited, but the inhibition magnitude was much less than that in Leghorn birds. It might be due to the specific downregulation of an important cooperator STIP1 Homology and U-Box Containing Protein 1 (STUB1) gene in this pathway in Fayoumi (Figure 10c). STUB1 is an E3 ubiquitin ligase that promoting protein degradation [29]. We speculate that due to the downregulation of STUB1, protein degradation in Fayoumis was reduced compared to Leghorn birds, which suggest that decreased protein degradation in liver may contribute to be more heat tolerant in Fayoumi line.

**Figure 11:**
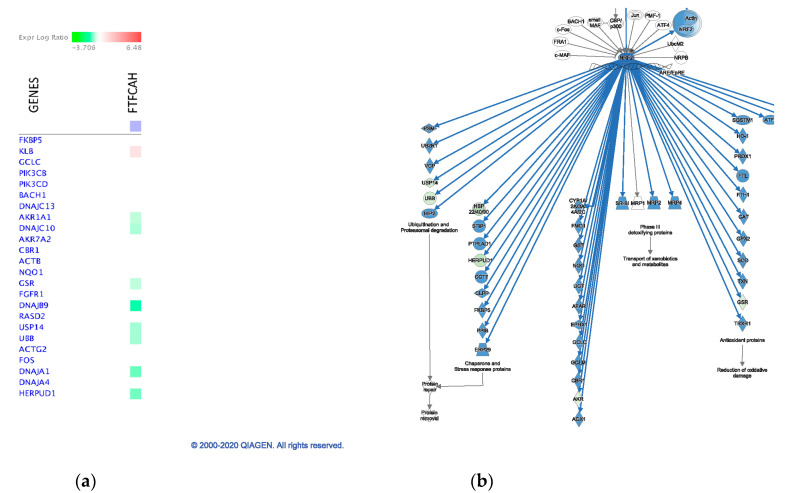
Partial of the NRF2-mediated Oxidative Stress Response pathway in Fayoumi birds with acute heat stress. (**a**) The heatmap of DEG in Fayoumis with acute heat stress matching to the pathway; (**b**) Molecule activity prediction of partial of the pathway with matching DEGs.

The inhibition of the NRF2-mediated Oxidative Stress Response was predicted in the Fayoumi line with AH as well (Figure 8c). Nuclear factor erythroid derived 2-related factor 2 (NRF2) is a master transcription regulator of antioxidant proteins that mediate cellular defense against oxidative stress [30] and a regulator of innate immunity by protecting from inflammatory stresses [31]. DEGs identified in Fayoumis at AH (1 up-regulated and 7 down-regulated) matched to this pathway and mainly contributed to repress “Ubiquitination and proteasomal degradation” and “Chaperone and Stress response” (Figure 11), which may reduce the protein degradation and relieve stress response in Fayoumis. Based on the preceding results, Fayoumis’ heat resilience may be due to their ability to indirectly inhibit protein degradation processes and alleviate cellular stressors during acute heat stress.

### 4.2. Host Response to Chronic Heat Stress Combined with NDV Infection

#### 4.2.1. The Leghorn Line

Slightly more DEGs were identified in Leghorns in the CH&NDV compared to the AH stage. But Leghorns still had far less DEGs than Fayoumis at the CH&NDV stage. Immune-related genes, such as MX1, Interferon α inducible protein 6 (IFI6) and Interferon induced protein with tetratricopeptide repeats 5 (IFIT5), were significantly upregulated with CH&NDV treatment (Table 8). They are all interferon related genes and important in host innate immune response [32,33,34].

Both immune and metabolism-related GO terms and pathways were significantly enriched by Leghorn up-regulated DEGs, while down-regulated DEGs only enriched metabolic functional terms. NDV infection under chronic heat stress did trigger Leghorn birds to activate their immune-related functions, however due to chronic heat stress, gene regulation of metabolic functions responding to heat stress was still predominant in the host response.

#### 4.2.2. The Fayoumi Line

Chronic heat stress and NDV infection stimulated a great number of DEGs in Fayoumi birds, which included both immune- and metabolic-related genes (Table 9). With over 500 up-regulated DEGs, significantly enriched GO terms and KEGG pathways were both immune and metabolism-related (Figure 7f). Results from canonical pathway prediction by IPA were highly supportive to the DAVID functional enrichment. Most of the immune-related pathways, such as the Role of RIG1-like receptors in antiviral innate immunity and NF-KB signaling pathway, were predicted to be activated (Supplementary file 5). Therefore, compared with Leghorns, Fayoumis had a more active antiviral immune response at CH&NDV.

On the other hand, most metabolic pathways, such as Oxidative phosphorylation and Cholesterol biosynthesis, had predicted inhibition (Supplementary File 5). Fifty-three down-regulated DEGs matched to the Oxidative phosphorylation pathway which led a very strong inhibition (Z = −7.28). During the process of oxidative phosphorylation, reactive oxygen species (ROS) will be generated at the electron transport chain [35]. Heat stress will induce an increase in ROS [36,37]. The excess ROS production is harmful and has negative effects on overall poultry performance as well as meat and egg quality [38]. Inhibition of this pathway maybe one of the specific protection mechanisms of Fayoumis to maintain the ROS homeostasis, which makes them more heat resilient. 

### 4.3. The Fayoumi Line had More Active Immune Response than the Leghorn Line with both Treatments

Genetic background plays a significant role in the resistance to heat stress and pathogens. Fayoumis were reported as a robust breed and more resistant to abiotic and several biotic stressors [25,39]. A previous genomic analysis study indicated that Fayoumi line had a stronger comprehensive immune system than the Leghorn line [40]. This has been supported by the results of our comparisons of these two lines.

When we looked at the functional terms and pathways significantly enriched by DEGs between genetic lines, Leghorn-biased genes were primarily involved in metabolic functions (Figure 6b,d,f,h) and Fayoumi-biased genes were involved in both immune and metabolic functions (Figure 6a,c,e,g). As an abiotic stressor, acute heat stress was able to trigger differential immune-related gene expression in Fayoumis but not in Leghorns.

This was consistent with the gene functional enrichment analysis. Immune-related functional terms were significantly enriched by both Leghorn and Fayoumi DEGs at CH&NDV. When we focused on the line and treatment specific DEGs (Figure 4 and Figure 5), Fayoumi specific DEGs had many immune-related functional terms significantly enriched. However, there were no immune-related functional terms identified by Leghorns specific DEGs (Supplementary files 3 and 4). Leghorn birds may be only able to activate a non-specific general host response to NDV infection during chronic heat stress. Gene regulation of metabolic functions responding to chronic heat stress was still predominant in host response in the Leghorn livers.

Meanwhile, we found that initiation of immune-related functions in response to heat stress was earlier in the Fayoumi line than the Leghorn line. The Negative regulation of glucocorticoid secretion and the Negative regulation of glucocorticoid receptor signaling pathway are two GO terms in the biological process that are both immune and metabolism-related. Glucocorticoids (GCs) are a group of steroid hormones that regulate a large number of immune and metabolic functions [41]. These two GO terms were not only significantly enriched by DEGs of Fayoumi birds at AH, but also enriched by DEGs of Leghorn birds at CH&NDV. This indicated that heat stress was not strong enough to initiate the immune response in Leghorn birds.

**Figure 12:**
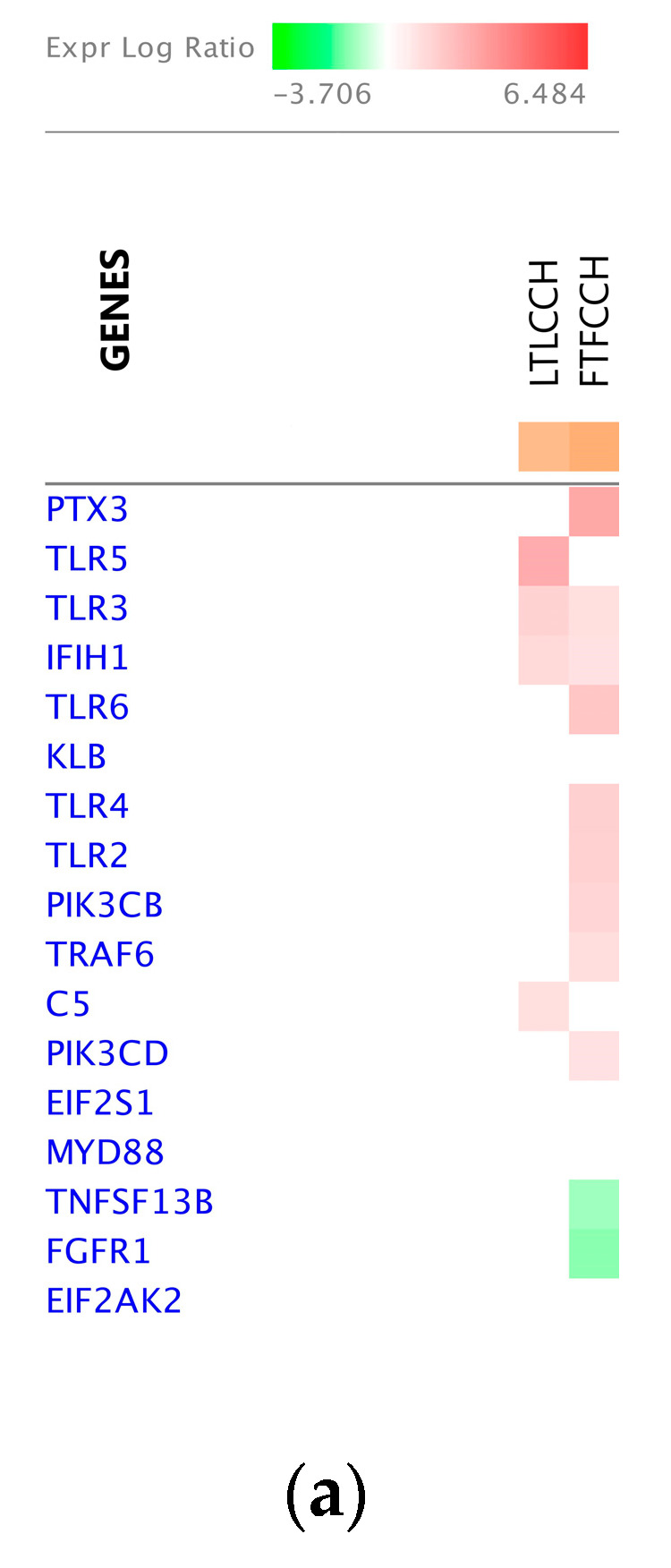

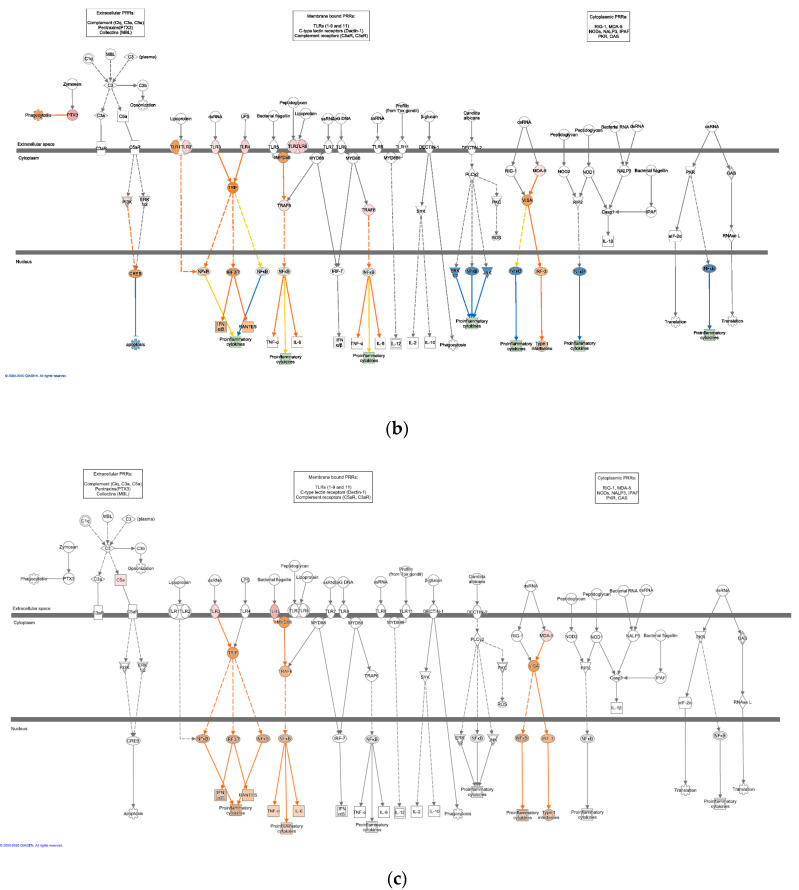
The Role of Pattern Recognition Receptors in Recognition of Bacteria and Viruses pathway in Fayoumi birds and Leghorn birds with NDV infection during chronic heat stress. (**a**) The DEG heatmap matching to the pathway; (**b**) The molecule activity prediction of the pathway in Fayoumi birds with NDV infection during chronic heat stress; (**c**) The molecule activity prediction of the pathway in Leghorn birds with NDV infection during chronic heat stress.

IPA analysis reinforced that Fayoumi birds had more or higher magnitude immune-related canonical pathways significantly enriched. With CH&NDV treatment, both Leghorn and Fayoumi birds activated the Role of Pattern Recognition Receptors in Recognition of Bacteria and Viruses pathway. However, with 10 DEGs matching to this pathway (Figure 12a), Fayoumi birds had a much higher magnitude of activation with strong inhibition of apoptosis and proinflammatory cytokine production (Figure 12b). Only 4 Leghorn DEGs matched to this pathway and these processes of Fayoumis were missing in Leghorns (Figure 12c).

In summary, Leghorn birds had an inconspicuous immune response compared to Fayoumis due to only regulating immune-related gene expression with the CH&NDV treatment. Fayoumi birds had a more active immune response than Leghorns by regulating immune-related gene expression with the AH treatment.

### 4.4. WGCNA Identified Potentially Important Gene Modules and Driver Genes

WGCNA can group genes into specific gene modules based on the correlations between genes and traits across all samples. Each gene module is a cluster of genes that share similar functions [15,20]. For those significantly correlated gene modules, some of them correlated with multiple factors (Table 4 and Table 5), which provided more insights regarding the treatment and line effects on physiological parameters.

#### 4.4.1. Significant Correlated Gene Modules

With AH, the Lightcyan module was the most positively correlated module with genetic lines. Genes highly expressed in this module were highly expressed in the Fayoumi line and significantly enriched “Calcium ion transmembrane import into michondrion” in the biological process (Supplementary file 7). Three genes, Single-pass membrane protein with aspartate rich tail1 (SMDT1), Mitochondrial calcium uniporter dominant negative β subunit (MCUB) and Mitochondrial calcium uptake 1 (MICU1), were mostly involved in this functional term, in which SMDT1 was the top 3 driver genes with very high GG (0.98) and MM (0.95). Fayoumi birds may maintain a stable cellular iCa^2+^ level by keeping high levels of these gene expression. Genes within this module such as SMDT1 could have great potential to be candidate genes for selection of genetic resistance to acute heat stress.

From our previous results, there was a reduction of Na^+^ and elevation of iCa^2+^ and Glu levels due to the acute heat stress in Leghorn birds [14]. This may be associated with gene expression in the Plum and Coral3 gene modules. Gene expression correlations were similar between iCa^2+^ and Glu, but opposite between these two and Na^+^ at AH. Meanwhile, the two modules also positively (Coral3) or negatively (Plum) correlated with pH, TCO_2_, HCO_3_ and BE. Identified driver genes such as Rho guanine nucleotide exchange factor 33 (ARHGEF33), Solute carrier family 14 member 1 (SLC24A1), Protease, serine 12 (PRSS12) and Calcium/calmodulin dependent protein kinase II inhibitor 1 (CAMK2N1) in the Coral 3 module and driver genes such as Tetratricopeptide repeat domain 30B (TTC30B), Heat shock protein 90 α family class B member 1 (HSP90AB1) and Mitogen-activated protein kinase 5 (MAP2K5) in the Plum module could play roles with respiratory alkalosis in Fayoumi and metabolic alkalosis in Leghorns.

With CH&NDV, the Black module was negatively correlated with genetic line. Genes highly expressed in this module had high expression levels in Leghorns and significantly enriched many metabolism-related GO terms and KEGG pathways. This support our previous findings that Leghorn birds had a stronger metabolic function. High abundance of driver genes in this module (Mitochondrial ribosomal protein L3 (MRPL3), 3-hydroxyanthranilate 3,4-dioxygenase (HAAO), G protein subunit α 11 (GNA11) and Succinate dehydrogenase complex flavoprotein subunit A (SDHA)) can be used as potential indicators of more heat susceptibility. 

#### 4.4.2. Other Potentially Important Driver Genes

As previously described, RDM1 and CHIA were always the top 1 and 2 genes for the gene modules positively correlated with Line for both treatments. Both of them had higher expression levels in the treated than the non-treated group. RDM1 can respond to mild heat shock in humans [42]. GO terms related to CHIA including carbohydrate binding and hydrolase activity [36,43]. CHIA also plays a role in T-helper cell type 2 immune response and contributes to the response of IL-13 in humans [44]. These two genes closely associated with both metabolic and immune response. At the same time, both had higher gene expression levels in Fayoumi birds than Leghorns which may partially contribute Fayoumis’ relative resistance to heat stress and NDV infection.

From the gene module analysis, gene expression levels in the Skyblue module at CH&NDV positively correlated with K^+^ and negatively correlated with Glu. Higher abundant genes in this module correlated with high K^+^ and low Glu levels. This support our previous phenotypical results that Leghorn birds had higher Glu and lower K^+^ levels with AH [14]. Blood Glu and K^+^ levels in chickens were negatively correlated with treatments in Leghorn birds (Correlation coefficient = 0.50) [14]. Solute carrier family 12 A5 (SLC12A5) and SLC26A9 were two drivers positively correlated with K^+^ levels and negatively correlated with Glu levels, respectively. The solute carrier family is a group of membrane transport proteins. SLC12A5 is one of the potassium/chloride transporters [45] and SLC26A9 was reported to transport chloride [46] and serve as an alternative chloride channel to regulate glucose metabolism in the pancreas [47]. These two membrane transporter genes may play important roles to regulate Glu and K^+^ levels in responding to heat stress and viral infection and are warranty for the further investigation.

## 5. Conclusions

Acute heat stress and chronic heat stress combined with NDV infection stimulated distinct physiological responses in the two highly inbred lines. The transcriptome analysis revealed that the Fayoumi line, more heat and NDV resistant, had many more DEGs than the Leghorn line during simultaneous chronic heat stress and NDV infection. Furthermore, immune regulation was triggered in Fayoumi birds with acute heat stress. Then Fayoumi birds continuously responded to heat stress and NDV infection by both immune and metabolic regulation. However, the Leghorn line, heat and NDV susceptible, at both AH and CH&NDV, mainly elicited a metabolic response. Moreover, metabolism regulation played a more important role in Leghorn birds when they were under heat stress even with viral infection. Potential candidate gene modules and driver genes identified in our current study complement the DGE list and allow for a better understanding of the functional relevance of these DEGs. Further investigation of these candidate genes, gene modules and interactive networks will be valuable for investigating the underline mechanisms for selection and breeding to improve the heat and pathogen resistance in chickens. 

## Figures and Tables

**Table 1:** Summary statistics of the RNA-seq data.

Line	Treatment	Time Point	Raw Reads	Aligned Reads	Alignment Rate
Leghorn	Non-treated	Acute Heat	199,693,733	183,292,871	91.79%
Leghorn	Treated	Acute Heat	187,612,958	170,964,578	91.13%
Fayoumi	Non-treated	Acute Heat	166,442,903	152,955,781	91.90%
Fayoumi	Treated	Acute Heat	195,204,499	178,279,792	91.33%
Leghorn	Non-treated	CH&NDV	106,510,459	98,128,436	92.13%
Leghorn	Treated	CH&NDV	120,871,158	111,479,701	92.23%
Fayoumi	Non-treated	CH&NDV	94,966,874	88,659,977	93.36%
Fayoumi	Treated	CH&NDV	115,633,054	108,848,582	94.13%

**Table 2:** Different gene expression regulation with the AH or CH&NDV treatment in Fayoumi birds.

Fayoumi DEG ^1^	Gene Description	AH ^2^ (log2foldchange)	CH&NDV ^3^ (log2foldchange)
*ZNFX1*	Zinc finger NFX1-type containing 1	−1.03	1.61
*USP18*	Ubiquitin specific peptidase 18	−1.64	1.22
*MX1*	MX dynamin like GTPase 1	−1.36	2.07
*CMPK2*	Cytidine/uridine monophosphate kinase 2	−1.49	1.68
*Novel gene*	Chromosome 3 open reading frame, human C8orf80	−1.19	1.24
*TLX1*	T cell leukemia homeobox 1	2.56	−1.87
*HSPB9*	Heat shock protein family B (small) member 9	1.59	−2.42
*FAM222A*	Family with sequence similarity 222 member A	1.35	−1.85

Note: ^1^ DEG, differentially expressed gene (FDR < 0.05); ^2^ AH, acute heat stress; ^3^ CH&NDV, chronic heat stress and NDV infection.

**Table 3:** Gene modules that were significantly associated with a factor (line or treatment or blood phenotype).

Factor	Positive Correlation		Negative Correlation	
	AH	CH&NDV	AH	CH&NDV
**Line ^1^**	Lightcyan (0.98 ^2^)	Paleturquoise (0.96)	Darkseagreen3 (−0.71)	Black (−0.76)
**Treatment**	Lightpink4 (0.74)	Bisque4 (0.85)	Blueviolet (−0.73)	Lightgreen(−0.60)
**pH**	Blueviolet (0.61)	Black (0.69)/ Lightgreen (0.69)	Plum (−0.73)	Darkolivergreen (−0.67)
**PCO_2_**	NS ^3^	NS	NS	NS
**HCO_3_**	Coral3 (0.59)	Lightgreen (0.54)/ Indianred4 (0.54)	Plum (−0.58)/ Lightpink4 (−0.58)	Coral2 (−0.56)
**TCO_2_**	Coral3 (0.60)	Indianred4 (0.61)	Plum (−0.57)/ Lightpink4 (−0.57)	Coral2 (−0.54)
**BE**	Coral3 (0.56)/ Blueviolet (0.56)	Lightgreen (0.69)	Plum (−0.66)	Coral2 (−0.66)
**Glu**	Coral3 (0.55)	NS	Lightcyan1 (−0.57)	Skyblue (−0.51)
**iCa^2+^**	Coral3 (0.51)	NS	NS	NS
**PO_2_**	Darkviolet (0.53)	Black (0.56)	NS	Paleturquoise (−0.77)
**sO_2_**	Darkviolet (0.54)	Black (0.65)	Lightcyan (−0.59)	Paleturquoise (−0.61)
**Na^+^**	Plum (0.57)	Plum1 (0.51)	Coral3 (−0.54)	NS
**K^+^**	Indianred4 (0.63)	Skyblue (0.53)	Antiquewhite2 (−0.59)	NS

Note: ^1^ To evaluate associations of co-expressed gene clusters with factors such as line and treatment, the Leghorn and Fayoumi lines were given nominal values of 1 and 2 and non-treated and treated the nominal values of 0 and 1; ^2^ Correlation coefficient of co-expressed gene clusters and factors; ^3^ No significant correlation.

**Table 4:** Gene modules that were significantly associated with factors (line, treatment or blood phenotypes) under the AH treatment.

	Module	Lightcyan	Plum	Lightpink4	Coral3	Blueviolet	Darkviolet
Factor							
**Line**		0.98 ^1^	0.69	0.26	−0.22	−0.52	−0.58
**Treatment**		−0.01	0.54	0.74	−0.23	−0.73	0.15
**pH**		−0.55	−0.73	−0.5	0.36	0.61	0.29
**PCO_2_**		0.34	0.19	−0.09	0.24	−0.15	−0.32
**TCO_2_**		−0.25	−0.57	−0.57	0.60	0.51	0.02
**HCO_3_**		−0.24	−0.58	−0.58	0.59	0.49	0.03
**BE**		−0.36	−0.66	−0.61	0.56	0.56	0.11
**PO_2_**		−0.43	−0.24	0.21	0.18	−0.05	0.53
**sO_2_**		−0.59	−0.58	−0.12	0.28	0.3	0.54
**Na^+^**		0.29	0.57	0.33	−0.54	−0.36	−0.34
**K^+^**		−0.27	0.077	0.45	−0.19	−0.02	0.33
**iCa^2+^**		−0.06	−0.38	−0.44	0.51	0.27	−0.05
**Glu**		−0.40	−0.55	−0.45	0.55	0.35	0.16

Note: ^1^ Correlation coefficient of co-expressed gene clusters and factors, positive values indicated positive correlations and negative values indicated negative correlations.

**Table 5:** Gene modules that were significantly associated with factors (line, treatment or blood phenotypes under the CH&NDV treatment.

	Module	Paleturquoise	Coral2	Skyblue	Indianred4	Lightgreen	Black
Factor							
**Line**		0.96 ^1^	0.62	0.32	0.28	−0.52	−0.76
**Treatment**		−0.15	0.59	−0.20	−0.15	−0.60	−0.28
**pH**		−0.37	−0.49	−0.14	−0.05	0.69	0.69
**PCO_2_**		0.07	−0.01	−0.21	0.45	−0.20	−0.24
**TCO_2_**		−0.26	−0.54	−0.41	0.61	0.44	0.40
**HCO_3_**		−0.31	−0.56	−0.42	0.54	0.54	0.48
**BE**		−0.42	−0.66	−0.42	0.50	0.69	0.67
**PO_2_**		−0.77	−0.29	−0.23	−0.46	0.21	0.56
**sO_2_**		−0.61	−0.39	−0.14	−0.38	0.45	0.65
**Na^+^**		0.35	0.17	0.10	0.37	−0.25	−0.45
**K^+^**		0.42	0.06	0.53	0.01	−0.26	−0.21
**iCa^2+^**		−0.23	−0.30	−0.44	0.44	0.24	0.26
**Glu**		−0.50	−0.42	−0.51	0.28	0.40	0.48

Note: ^1^ Correlation coefficient of co-expressed gene clusters and factors, positive values indicated positive correlations and negative values indicated negative correlations.

**Table 6:** Top five driver genes for gene modules that were significantly associated with a factor (line, treatment or blood phenotypes) for the AH treatment.

Factor	Correlation	Module Color	Gene Name (GS ^1^, MM ^2^)
**Line**	Positive	Lightcyan	*RDM1* (1.00,0.98), *CHIA* (0.99,0.95), *SMDT1* (0.98,0.95), *IFT57* (0.98,0.96), *ERMP1* (0.98,0.95)
	Negative	Darkseagreen3	*FAXDC2* (0.81, −0.93), *FMO3* (0.80, −0.95), *CYP2J21* (0.80, −0.91), *PLXNA4* (0.79, −0.85), *KLHL8* (0.78, −0.85)
**Treatment**	Positive	Lightpink4	*BTBD7* (0.80, 0.67), *MCF2* (0.78, 0.63), *PLD1* (0.78, 0.82), *GRIP2* (0.75, 0.64), *CECR2* (0.75, 0.80)
	Negative	Blueviolet	*CACNG5* (0.95, −0.65), *ERLEC1* (−0.91, 0.82), *PSMC1* (−0.90, 0.88), *EIF2A* (−0.90, 0.77), *HSPA5* (−0.89, 0.86)
**pH**	Positive	Blueviolet	*gga-mir−1723* (0.72, 0.81), *NEMF* (0.72, 0.79), *HSP90AA1* (0.71, 0.93), *SF3B3* (0.71, 0.71), *VKORC1L1* (0.71, 0.92)
	Negative	Plum	*TTC30B* (−0.85, 0.81), *ENSGALG00000040869* (−0.80, 0.88), *PPCDC* (−0.79, 0.88), *HSP90AB1* (0.78, −0.88), *TTC17* (−0.77, 0.88)
**HCO_3_**	Positive	Coral3	*ENSGALG00000045967* (0.76, 0.73), *ARHGEF33* (0.71, 0.88), *SLC24A1* (0.67, 0.63), *PRSS12* (0.66, 0.50), *SPTAN1* (0.64, 0.65)
	Negative	Lightpink4	*TGS1* (0.75, −0.82), *UPF3B* (0.73, −0.94), *ZBTB8OS* (0.73, −0.79), *SRSF2* (0.73, −0.86), *gga-mir−6585* (0.72, −0.86)
		Plum	*ENSGALG00000007624* (0.82, −0.56), *TTC30B* (−0.76, 0.81), *BARX2* (−0.71, 0.70), *RF00392* (0.70, −0.77), *NLE1* (0.69, −0.79)
**TCO_2_**	Positive	Coral3	*ARHGEF33* (0.70, 0.88), *SLC24A1* (0.64, 0.63), *CFAP58* (0.63, 0.78), *PRSS12* (0.63, 0.50), *SPTAN1* (0.62, 0.65)
	Negative	Lightpink4	*TGS1* (0.76, −0.82), *ZBTB8OS* (0.74, −0.79), *SRSF2* (0.72, −0.86), *UPF3B* (0.71, −0.94), *GTF2B* (0.70, −0.81)
		Plum	*ENSGALG00000007624* (0.78, −0.56), *TTC30B* (−0.75, 0.81), *RF00392* (0.71, −0.77), *BARX2* (−0.70, 0.70), *NLE1* (0.68, −0.79)
**BE**	Positive	Coral3	*ENSGALG00000045967* (0.79, 0.73), *PRSS12* (0.75, 0.50), *ARHGEF33* (0.73, 0.88), *SPTAN1* (0.71, 0.65), *SLC24A1* (0.70, 0.63)
		Blueviolet	*INSIG1* (0.76, 0.65), *SUCO* (0.74, 0.59), *ENOX2* (0.72, 0.73), *NABP1* (0.69, 0.86), *USP14* (0.69, 0.94)
	Negative	Plum	*TT30B* (−0.85, 0.81), *ENSGALG00000007624* (0.80, −0.56), *PPCDC* (−0.73, 0.88), *NLE1* (0.72, −0.79), *RF00392* (0.71, −0.77)
**Glu**	Positive	Coral3	*CFAP58* (0.58, 0.78), *ENSGALG00000045967* (0.57, 0.73), *CAMK2N1* (0.57, 0.72), *ENSGALG00000035761* (0.54, 0.78), *ENSGALG00000040923* (0.53, 0.68)
	Negative	Lightcyan1	*ST6GALNAC3* (−0.82, 0.65), *COL20A1* (−0.82, 0.69), *SLC10A4* (0.67, 0.68), *MYOM2* (−0.66, 0.81), *HTR7L* (−0.60, 0.80)
**iCa^2+^**	Positive	Coral3	*ENSGALG00000035761* (0.60, 0.78), *SPTAN1* (0.58, 0.65), *ENSGALG00000045967* (0.52, 0.73), *ENSGALG00000011252* (0.51, 0.91), *CAMK2N1* (−0.55, 0.51)
**PO_2_**	Positive	Darkviolet	*VEZF1* (0.76, 0.59), *CDON* (0.76, 0.80), *ENSGALG00000028262* (0.75, 0.81), *CNGA2* (0.72, 0.58), *MN1* (0.72, 0.83)
**sO_2_**	Positive	Darkviolet	*DDR2* (0.76, 0.77), *CNGA2* (0.75, 0.58), *ENSGALG00000026460* (0.72, 0.71), *MAPK8IP1* (0.72, 0.88), *KLF10* (0.71, 0.57)
	Negative	lightcyan	*TRIM37* (−0.82, 0.80), *SEPTIN10* (−0.81, 0.87), *C11H16ORF87* (−80, 0.77), *ACE2* (−0.79, 0.84), *BLOC1S4* (−0.78, 0.88)
**Na^+^**	Positive	Plum	*MXD4* (0.81, 0.75), *MID1* (0.75, 0.88), *BARX2* (0.74, 0.70), *TTC30B* (0.73, 0.81), *MAP2K5* (0.66, 0.84)
	Negative	Coral3	*ENSGALG00000045967* (−0.79 0.73), *FAM81A* (0.72, −0.83), *ARHGEF33* (−0.66, 0.88), *ENSGALG00000040923* (−0.65, 0.68), SLC24A1 (−0.64, 0.63)
**K^+^**	Positive	Indianred4	*CEMIP* (0.75, 0.92), *FAM189A2* (0.74, 0.81), *ELK3* (0.72, 0.76), *TMEM204* (0.71, 0.94), *ALPL* (0.71, 0.86)
	Negative	Antiquewhite2	*KCNMB4* (0.77, −0.79), *HEATR6* (−0.75, 0.59), *UBE2T* (−0.73, 0.68), *UNC93A* (−0.69, 0.83), *FLRT3* (−0.60, 0.51)

Note: ^1^ Gene Significance, ^2^ Module Membership.

**Table 7:** Top five driver genes for gene modules that were significantly associated with a factor (line, treatment or blood phenotypes) for CH&NDV treatment.

Trait	Correlation	Module Color	Gene Name (GS ^1^, MM ^2^)
**Line**	Positive	Paleturquoise	*RDM1* (0.99, 0.95), *CHIA* (0.99,0.95), *DPT* (0.98,0.94), *GALNT16* (0.98, 0.93), *COMTD1* (0.97, 0.93)
	Negative	Black	*MRPL3* (−0.90, 0.87), *HAAO* (−0.88, 0.88), *LPAR2* (0.86, −0.91), *GNA11* (−0.85, 0.90), *ENSGALG00000041512* (0.85, −0.77)
**Treatment**	Positive	Bisque4	*FAM46A* (0.92, 0.81), *DTX3L* (0.92, 0.81), *ARNTL* (0.91, 0.77), *ENSGALG00000042001* (0.90, 0.79), *MX1* (0.89, 0.78)
	Negative	Lightgreen	*PSMD1* (−0.89, 0.66), *PNPLA7* (−0.89, 0.70), *IFIH1* (0.83, −0.80), *LCAT* (−0.82, 0.84), *HBA1* (−0.81, 0.82)
**pH**	Positive	Black	*COX7A2L* (0.82, 0.88), *CCDC25* (0.81, 0.86), *ND4L* (0.79, 0.91), *RAB11A* (0.79, 0.79), *TIMM44* (0.79, 0.88)
		Lightgreen	*ENSGALG00000034218* (0.87, 0.90), *COMMD9* (0.87, 0.88), *ENSGALG00000042254* (0.85, 0.89), *TPM3* (0.85, 0.86), *ISCU* (0.84, 0.87)
	Negative	Darkloivergreen	*PRIMPOL* (−0.84, 0.79), *HELB* (−0.83, 0.92), *ENSGALG00000045606* (−0.82, 0.86), *INTS6* (−0.81, 0.88), *PCF11* (−0.81, 0.90)
**HCO_3_**	Positive	Lightgreen	*ZP4* (0.73, 0.55), *HS6ST1* (0.72, 0.87), *RBP3* (0.72, 0.57), *HIST1H46* (0.72, 0.87), *PCCB* (0.70, 0.86)
		Indianred4	*TNNC2* (0.68, 0.77), *ZP2* (0.62, 0.56), *ENSGALG00000026655* (0.61, 0.98), *ENSGALG00000041238* (−0.61, −0.81), *ENSGALG00000012416* (−0.61, −0.76)
	Negative	Coral2	*TMEM154* (−0.66, 0.90), *FAH* (0.66, −0.93), *MGARP* (0.64, −0.92), *AAMDC* (0.61, −0.89), *FANCL* (0.61, −0.86)
**TCO_2_**	Positive	Indianred4	*TNNC2* (0.64, 0.77), *ENSGALG00000026655* (0.62, 0.98), *CCN3* (0.61, 0.69), *ENSGALG00000029695* (0.60, 0.99), *ST6GAL2* (0.58, 0.67)
	Negative	Coral2	*PNPLA3* (−0.70, 0.82), *TMEM154* (−0.69, 0.90), *CYP1AC1* (0.65, −0.70), *REEP2* (−0.63, 0.82), *MANBAL4* (−0.62, 0.73)
**BE**	Positive	Lightgreen	*HS6ST1* (0.82, 0.87), *HIST1H46* (0.80, 0.87), *CPS1* (0.80, 0.85), *SNX3* (0.80, 0.62), *MALSU1* (0.80, 0.80)
	Negative	Coral2	*MGARP* (0.79, −0.92), *AAMDC* (0.77, −0.89), *HADHA* (0.76, −0.92), *FBXL18* (−0.76, 0.80), *REEP2* (−0.74, 0.82)
**Glu**	Negative	Skyblue	*INAVA* (−0.69, 0.71), *ENSGALG00000007007* (−0.69, 0.75), *PTHLH* (−0.66, 0.66), *SLC12A5* (−0.64, 0.79), *SINHCAF* (−0.64, 0.60)
**PO_2_**	Positive	Black	*RAB31* (0.78, 0.60), *GAN11* (0.71, 0.81), *SEPTIN6* (0.71, 0.62), *CRADD* (0.69, 0.71), *POLR3F* (0.67, 0.76)
	Negative	Paleturquoise	*VAMP7* (0.89, −0.63), *BMP2* (−0.87, 0.91), *AP3B1* (0.85, −0.78), *NLN* (0.84, −0.83), *C3orf33* (−0.84, 0.75)
**sO_2_**	Positive	Black	*GAN11* (0.79, 0.90), *SEPTIN6* (0.78, 0.75), *RAB31* (0.77, 0.68), *NEK4* (0.74, 0.90), *ENSGALG00000037200* (0.72, 0.75)
	Negative	Paleturquoise	*VAMP7* (0.91, −0.63), *MGAT4D* (−0.85, 0.73), *ABCA4* (−0.80, 0.71), *ADAMTS3* (−0.79, 0.81), *TRMT61B* (−0.78, 0.80)
**Na^+^**	Positive	Plum1	*CNN1* (0.68, 0.70), *GRK6* (0.66, 0.63), *ENSGALG00000034399* (0.66, 0.87), *BAHCC1* (0.65, 0.91), *SLC7A3* (0.65, 0.78)
**K^+^**	Positive	Skyblue	*RF00569* (0.79, 0.65), *CACNG2* (0.61, 0.77), *SLC26A9* (0.57, 0.85), *SCRT1* (0.55, 0.63), *ZPLD1* (0.55, 0.52)

Note: ^1^ Gene Significance, ^2^ Module Membership.

**Table 8:** Top 10 up- and down-regulated DEGs in the Leghorn line.

Treatment	Gene Name	Gene Description	log2 Fold Change
**AH**	*GADD45G*	Growth arrest and DNA damage inducible γ	1.91
	*TRPC3*	Transient receptor potential cation channel subfamily C member 3	1.8
	*GPRC5B*	G protein-coupled receptor class C group 5 member B	1.63
	*MSC*	Musculin	1.58
	*CEMIP*	Cell migration inducing hyaluronidase 1	1.53
	*GPR182*	G protein-coupled receptor 182	1.48
	*ART7C*	Erythroblast NAD--arginine ADP-ribosyltransferase-like	1.43
	*ADAMTS15*	ADAM metallopeptidase with thrombospondin type 1 motif, 15	1.38
	*DNASE2B*	Deoxyribonuclease 2 β	1.36
	*LDLRAD3*	Low density lipoprotein receptor class A domain containing 3	1.31
	*SOCS1*	Suppressor of cytokine signaling 1	−1.43
	*BAG3*	BCL2 associated athanogene 3	−1.48
	*TTC7A*	Tetratricopeptide repeat domain 7A	−1.52
	*DNAJA4*	DnaJ heat shock protein family (Hsp40) member A4	−1.56
	*JMJD6*	Jumonji domain containing 6	−1.57
	*HSPH1*	Heat shock protein family H (Hsp110) member 1	−1.67
	*HSP90AA1*	Heat shock protein 90 α family class A member 1	−1.96
	*HSPA2*	Heat shock 70kDa protein 2	−2.25
	*HSPA5*	Heat shock 70kDa protein 5 (glucose-regulated protein, 78kDa)	−2.39
	*HSPA8*	Heat shock 70kDa protein 8	−2.84
**CH&NDV**	*NSUN7*	NOP2/Sun RNA methyltransferase family member 7	6.48
	*HSPB2*	Heat shock protein family B (small) member 2	4.38
	*IFI6*	Interferon α inducible protein 6	3.62
	*OASL*	2’−5’-oligoadenylate synthetase like	3.32
	*IFIT5*	Interferon induced protein with tetratricopeptide repeats 5	3.31
	*MX1*	MX dynamin like GTPase 1	3.28
	*ADGRG2*	Adhesion G protein-coupled receptor G2	3.24
	*CNN1*	Calponin 1	3.06
	*LRRC56*	Leucine rich repeat containing 56	2.8
	*CLCA1*	Chloride channel accessory 1	2.71
	*RPL3L*	Ribosomal protein L3 like	−1.64
	*ABHD6*	Abhydrolase domain containing 6	−1.73
	*BHLHE40*	Basic helix-loop-helix family member e40	−1.75
	*CYP2C18*	Cytochrome P450 family 2 subfamily C member 18	−1.77
	*EPB42*	Erythrocyte membrane protein band 4.2	−1.82
	*ANGPTL4*	Angiopoietin like 4	−1.93
	*LMBR1L*	Limb development membrane protein 1 like	−2.31
	*CASP14*	Caspase 14, apoptosis-related cysteine peptidase	−2.34
	*ACOT12*	Acyl-CoA thioesterase 12	−2.35
	*DIO2*	Iodothyronine deiodinase 2	−3.71

**Table 9:** Top 10 up- and down-regulated DEGs in the Fayoumi line.

Treatment	Gene Name	Gene Description	log2 Fold Change
**AH**	*CLRN2*	Clarin 2	6.18
	*ZBTB16*	Zinc finger and BTB domain containing 16	3.46
	*TLX1*	T cell leukemia homeobox 1	2.56
	*GRIP2*	Glutamate receptor interacting protein 2	2.42
	*CCDC103*	Coiled-coil domain containing 103	2.18
	*MBOAT2*	Membrane bound O-acyltransferase domain containing 2	1.89
	*PPARGC1A*	PPARG coactivator 1 α	1.85
	*TMEM255A*	Transmembrane protein 255A	1.8
	*GPRC5B*	G protein-coupled receptor class C group 5 member B	1.78
	*HKDC1*	Hexokinase domain containing 1	1.76
	*PTPRD*	Protein tyrosine phosphatase, receptor type D	−1.53
	*USP18*	Ubiquitin specific peptidase 18	−1.64
	*TTC7A*	Tetratricopeptide repeat domain 7A	−1.79
	*HSPA8*	Heat shock 70kDa protein 8	−1.88
	*SCF14M2*	Solute carrier family 14 member 2	−1.92
	*HSP90AA1*	Heat shock protein 90 α family class A member 1	−1.96
	*FBN2*	Fibrillin 2	−2.16
	*HSPA5*	Heat shock 70kDa protein 5 (glucose-regulated protein, 78kDa)	−2.3
	*BHLHA15*	Basic helix-loop-helix family member a15	−3.12
	*PRRX1*	Paired related homeobox 1	−3.2
**CH&NDV**	*G0S2*	Gallus gallus G0/G1 switch 2 (G0S2), mRNA	4.9
	*LAMB3*	Laminin subunit β 3	4.48
	*NPAS2*	Gallus gallus neuronal PAS domain protein 2 (NPAS2), mRNA	4.03
	*PLCH1*	Phospholipase C eta 1	3.11
	*TMEM255A*	Transmembrane protein 255A	3.02
	*MYBPC3*	Myosin binding protein C, cardiac	2.97
	*PROK2*	Prokineticin 2	2.96
	*SCNN1B*	Sodium channel epithelial 1 β subunit	2.86
	*SERPINB10B*	Gallus gallus serpin peptidase inhibitor, clade B, member 10 B	2.84
	*PTX3*	Pentraxin 3	2.79
	*E3UPLT*	E3 ubiquitin-protein ligase Topors-like	−2.85
	*PLCE1*	Phospholipase C epsilon 1	−3.08
	*NKX2-3*	NK2 homeobox 3	−3.41
	*APOV1*	Apovitellenin 1	−3.53
	*DIO2*	Iodothyronine deiodinase 2	−3.64
	*SPARCL1*	SPARC like 1	−3.68
	*CHAC1*	ChaC glutathione specific γ-glutamylcyclotransferase 1	−4.16
	*NOV*	Nephroblastoma overexpressed (NOV), mRNA	−4.76
	*EDSC*	Epidermal differentiation protein rich in serine and cysteine, mRNA	−5.19
	*NKX6-3*	NK6 homeobox 3	−6.76

## Supplementary Materials

The following are available online at https://www.mdpi.com/2073-4425/11/9/1067/s1, Supplementary file 1 -DEGs identified in between-line comparisons. Supplementary file 2 -DEGs identified in within-line comparisons. Supplementary file 3 -GO terms for between-line specific DEGs. Supplementary file 4 -GO terms for within-line specific DEGs. Supplementary file 5 -Canonical pathways predicted by DEGs in within-line comparisons. Supplementary file 6 -Top 5 Driver genes for each gene module from WGCNA. Supplementary file 7 -GO terms for all significantly correlated gene modules.
